# Life Cycle Assessment of an Avocado: Grown in South Africa—Enjoyed in Europe

**DOI:** 10.1007/s00267-024-02009-w

**Published:** 2024-06-27

**Authors:** Sheldon A. Blaauw, André Broekman, James W. Maina, Wynand J. v. d. M. Steyn, William A. Haddad

**Affiliations:** 1 Arup, East West Building, 1 Tollhouse Hill, Nottingham, NG1 5AT UK; 2 Department of Civil Engineering, University of Pretoria, Private Bag X20, Hatfield, 0028 South Africa; 3 Zutari, Riverwalk Office Park, 41 Matroosberg Road, Ashlea Gardens, Pretoria, 0081 South Africa; 4 Ecosystem Services, ZZ2 Group, P.O. Box 19, Mooketsi, Limpopo 0825 South Africa

**Keywords:** Life cycle assessment, Agriculture, Carbon dioxide equivalent, Avocado farming, Climate change

## Abstract

Food production is known to have significant environmental impacts, with the main contributors residing in the farming and transportation life cycle phases. Of the various food products transported around the world, avocados have increasingly gained attention as a high-commodity superfood. Avocados require specific climatic and agricultural conditions for farming, with the most fertile land and conditions located outside Europe. Consequently, most avocados consumed in Europe are imported over vast geographical distances, with little information available to quantify the environmental impacts of this imported superfood. This paper aims to present the most detailed life cycle assessment results of an avocado cultivated, grown and harvested in the Limpopo Province of South Africa and exported to the European market for sale and consumption. A life cycle assessment was developed for the farming, harvesting, handling, packaging, ripening, transportation, and carbon sequestration potential of the avocado, and it was used to conduct a holistic life cycle assessment. Input data was obtained through an 18-month data collection campaign across the relevant stakeholders. A baseline ‘business-as-usual’ scenario is focused on throughout this study, and scope for optimisation is identified for each life cycle phase where applicable, accompanied by uncertainty analyses. Results show a total carbon input of 904.85 kg CO_2_e/tonne. Mitigating this, 521.88 kg CO_2_e/tonne is offset, resulting in a net carbon footprint of 382.97 kg CO_2_e/tonne with uncertainty ranges of −23.22 to +58.69 kg CO_2_e/tonne, normalised to 57.45 g CO_2_e/avocado grown in South Africa and sold in Europe. The environmental impacts of the avocado industry under consideration are largely mitigated by the “nature first” philosophy of the farming and logistics enterprises, which have made significant investments in reducing emissions. Sensitivity analyses indicate that implementing large-scale renewable energy, using alternative packaging instead of cardboard, and selling avocados unripened could further enable the farming enterprise to achieve Net Zero objectives. These measures could reduce baseline emissions from 382.97 kg CO_2_e/tonne to a theoretical −68.54 kg CO_2_e/tonne, representing a 117.9% decrease. Although this study does not quantify climate change impacts, qualitative analyses suggest that climate change will have a net negative effect on the avocado industry in South Africa. These regions, typically located in micro-climates, are projected to become wetter and warmer, adversely affecting crop phenology, pest control, road conditions, management complexity, farmer livelihoods, and food security. The study recommends large-scale implementation of the optimisation strategies identified to achieve Net Zero objectives and the development of proactive climate change mitigation strategies to enhance the resilience of avocado supply chains to future stressors. These insights are crucial for policymakers, industry stakeholders, and consumers aiming to promote sustainability in the avocado market.

**Graphical Abstract:**

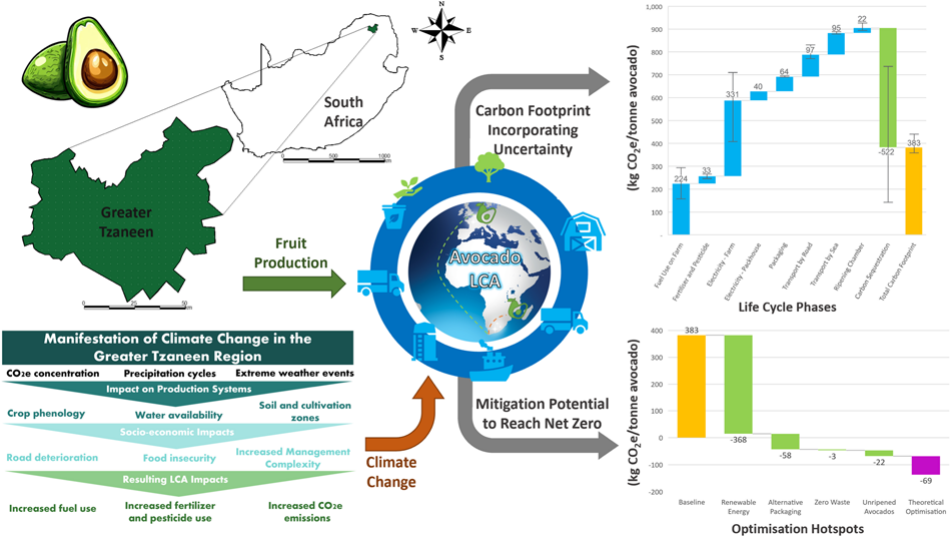

## Introduction

Recently, the University of Pretoria has undertaken comprehensive research projects to investigate the postharvest condition of fresh produce in South Africa, emphasising tomatoes and avocados. This investigation includes the handling of the fresh produce in packhouse environments and the interconnected transportation thereafter across vast distances by road, first, from Tzaneen in Limpopo Province to Cape Town in Western Cape Province, which is approximately ±1800 km and then by sea from Cape Town to Rotterdam in the Netherlands, which is ~±12,500 km, destined for international markets. The transportation value chain of fresh produce such as avocados is of significance to not only the farmers, but all stakeholders involved in successfully executing the underlying operations of the various phases, including local citizens and the surrounding communities who rely on the industry for their livelihoods.

In the wake of increasing regulatory requirements and consumers’ awareness of sustainable practices, proposals for carbon taxes to promote greener energy sources and fragile freight industries amplified by disruptions from global events (pandemics, armed conflicts and energy shortages), the need to quantitatively evaluate the sustainability and environmental impact of highly-valued avocadoes has received renewed focus. However, there is little to no information available that accounts for the total energy input requirements and environmental impact (equivalent CO_2_ emissions) related to the avocado industry, particularly on a per-avocado basis. This study is aimed at generating a Life Cycle Assessment (LCA) for the farming, harvesting, handling, packaging, artificial ripening, transportation, and carbon sequestration potential of a typical avocado grown in South Africa destined for European markets. Input data was obtained through an 18-month data collection campaign across the relevant stakeholders.

## Critical Appraisal of Existing Avocado Life Cycle Assessment Research

Many studies have been conducted to estimate the environmental impacts of avocados (Stoessel et al. 2012; Graefe et al. 2013; Astier et al. 2014; Bell et al. 2018; Solarte-Toro et al. 2022; Majumdar and McLaren 2023) with wide-ranging LCA methodologies applied producing varying results.

Astier et al. (2014) delved into Mexico’s energy usage and greenhouse gas emissions of organic and conventional avocado orchards. The methodology involved selecting representative avocado orchards, collecting data through interviews and schedules, and computing greenhouse gas emissions using established factors. The findings revealed a heavy reliance on fossil fuel inputs, machinery, and nitrogen fertilisers in both systems, with no significant disparity in energy consumption between them. Graefe et al. (2013) assessed the resource consumption, greenhouse gas emissions, and potential role in climate change mitigation of various tropical fruit species, including avocados, grown in Colombia. Similarly, Bell et al. (2018) investigated the challenges confronting California’s agricultural sector amidst extreme hydrological events. They underscored the necessity of exploring alternative irrigation water sources, focusing on carbon and energy considerations. Bartl et al. (2012) conducted LCAs on agricultural production in Peru, offering valuable decision support for local farmers and authorities by examining various crop combinations and irrigation systems for future development, whose methodology was equally applied by Majumdar and McLaren (2023) to investigate the orchard phase of the New Zealand avocado value chain, pinpointing key areas for improvement efforts.

A critical appraisal of this research reveals several key trends and gaps in understanding the environmental footprint of avocado production. Regarding the goal and scope, most studies seek to quantify and compare energy use and greenhouse gas emissions with select studies broadening the impact categories to include consideration of water use, agricultural land occupation (Bartl et al. 2012), labour, and ecotoxicity (Bell et al. 2018; Stoessel et al. 2012); acknowledging the challenges in accurately quantifying latter factors due to uncertainties in upstream processes. The cradle-to-gate or farm-to-fork system boundaries are commonly applied. Similarly, a functional unit—one tonne of product with a reference flow of one hectare—is generally utilised (Du Plessis et al. 2022; Majumdar and McLaren 2023).

Data collection methods employed to gather information relevant to environmental assessments of avocado production are diverse, ranging from methods which include primary data collection from farms or producing companies (Esteve-Llorens et al. 2022), as well as secondary data from agricultural databases and literature (Du Plessis et al. 2022). Select studies (Astier et al. 2014; Majumdar and McLaren 2023) relied on surveys to capture data, emphasising obtaining localised and detailed data to accurately assess environmental impacts, ensuring data quality and reliability through validation and verification processes.

Notably, carbon sequestration—an important aspect of the carbon balance within production systems—was notably absent from consideration across most studies (Stoessel et al. 2012; Frankowska et al. 2019; Solarte-Toro et al. 2022). Additionally, application of sensitivity and uncertainty analyses varies among studies, with some explicitly conducting these analytical techniques (Bendotti Avocado, 2021; Bell et al. 2018; Esteve-Llorens et al. 2022) while most do not. In the realm of sensitivity analyses within LCA studies, a prevalent method involves altering one input parameter individually while holding others constant, known as the One-factor-at-a-time approach. This method pinpoints the most influential parameters (Wei et al. 2015). Various techniques are commonly employed concerning uncertainty analyses, including parameter-, model-, scenario-, or data quality assurance-uncertainty. These methods often utilise Monte Carlo simulations to ascertain lower and upper quartile values, visualising uncertainty in the process (Bamber et al. 2019).

Overall, addressing these trends and gaps in future research efforts is crucial for enhancing the accuracy and comprehensiveness of avocado sustainability assessments.

## Materials and Method

### Goal and Scope

This study endeavours to conduct a comprehensive LCA of a representative South African avocado exported to international markets while laying the groundwork for subsequent research studies. Beyond mere assessment, an overarching objective is to delineate a methodological framework that facilitates the replication of LCAs for avocados and a diverse array of fruits, vegetables, and food products. The study’s scope, characterised as an extensive desktop study, aims to amalgamate disparate data from industry experts such as ZZ2 and H&S (Halls and Sons), recent audit data, and literature-derived information about energy, fuel, water consumption, consumables, land usage change, and industry best practices.

In addition to fulfilling immediate objectives, this research aspires to contribute to broader scientific pursuits. It furnishes a sensitivity analysis for optimisation and heightened sustainability measures, an uncertainty analysis to assess results variance, and a replicable methodology for subsequent studies. The inclusivity of data within the LCA model is meticulously guided by consultations with subject matter experts, ensuring a comprehensive understanding of diverse processes. Furthermore, the study’s inclusive scope encompasses field studies, requisite measurements, and instrumentation demands, thereby establishing a pivotal precedent for systematic assessments within agricultural product life cycles.

**Fig. 1:**
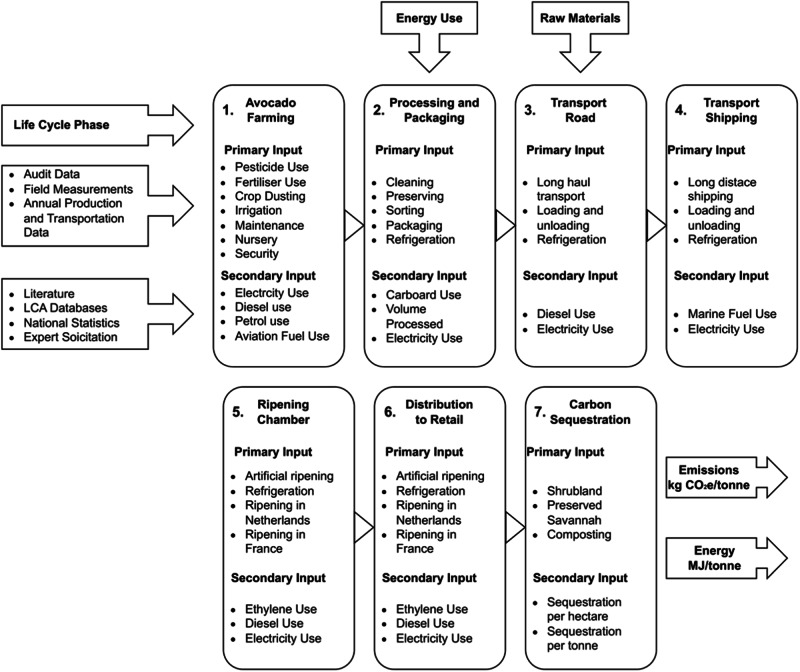
Life Cycle of an Avocado Considered in This Study

This study adopts a one-tonne product of avocados as the functional unit for a comprehensive farm-to-fork analysis. Data were collected for a reference flow of one hectare of mature avocado orchards (>70% production per hectare), used to calculate the results for one-tonne product and subsequently on a per avocado basis. The system boundaries of this study encompass all processes involved in the production and distribution of exported avocados grown on mature plantations while excluding avocados sold locally within South Africa, as shown in Fig. 1. A matrix explicitly developed for South African studies, based on ISO 14040 guidelines (ISO 2006), is utilised to assess data quality through Data Quality Indicators (DQIs) provided in the main body text with specific details regarding DQI reference and how scores are derived given in Table S34 in the supplementary information. Primary data was used for input types and quantities to all foreground processes. In contrast, secondary data (derived from literature) were used for background processes (e.g. extraction of raw materials, production of fertilisers, etc.), representing average technology used globally/regionally.

#### Key Assumptions

Key assumptions, informed by consultations with stakeholders and practical production rates, are as follows: avocados weigh an average of 150 g (100 g flesh), transported at 160,000 avocados per twenty-foot-equivalent (TEU) container (24 tonnes/TEU); farm wastage is 6.74%, with an additional 3% estimated for transportation; cartons contain 27 avocados at ~4.4 kg total weight; one hectare yields 9.5 metric tonnes annually, and avocado calorie count is 134 kcal per 100 g flesh. The Smart Avocado (smAvo) (Broekman et al. 2020) measured additional details, like transportation distances and speeds.

### Impact Categories and Assessment Methods

This study’s assessment of environmental impacts encompasses various key indicators, with a primary focus on energy use and CO_2_e emissions. These indicators are pivotal in understanding the avocado supply chain’s carbon footprint and energy efficiency, reflecting critical aspects of environmental sustainability. Additionally, consideration is given to other impact categories, such as water use and social emissions, albeit selectively, contingent upon data availability and aligned study objectives. Assessment methods employed in this study integrate quantitative analysis of resource consumption and emissions, utilising LCA methodologies to systematically evaluate the avocado supply chain’s environmental performance. These methods facilitate the identification of hotspots and optimisation opportunities discussed in later sections, guiding informed decision-making towards enhancing the overall sustainability of avocado production and distribution.

### Data Collection Strategy and Quality Requirements

This study employed a comprehensive data collection approach, utilising stakeholder engagement and tracking technology to trace the avocado journey. An 18-month campaign led by diverse stakeholders gathered data, including audit information from Blue North Sustainability, covering fuel, fertiliser, pesticide, and electricity usage across 21 plantations. For this study, 10 of these plantations were selected based on their alignment with the specified goal and scope criteria, mainly focusing on mature plantation sites. Stringent protocols ensured data quality, with industry experts and logistics companies providing additional validation. The amalgamation of industry expertise and advanced tracking methods ensures a robust dataset, reflecting a thorough and transparent approach across different life cycle phases.

### Carbon Sequestration Methodology

The carbon sequestration potential for avocados was calculated by evaluating the contributions from three main sources: composting dairy manure and green waste, shrubland, and preserved savannah. The calculations involved several steps to convert these contributions into a per-tonne-of-avocado basis. Firstly, literature values for carbon sequestration potential from various sources such as compost, shrubland, and savannah are utilised. Secondly, the total area or volume used is computed by determining the quantity of compost employed on the farm while also considering wastage, along with identifying the total area dedicated to shrubland and savannah on the farm. Thirdly, proportions are allocated to avocado cultivation by utilising farm-specific factors to ascertain the proportion of shrubland and savannah areas linked to avocado cultivation. Finally, to standardise the analysis, the data is converted to per hectare and per tonne basis. This involves calculating the contributions of each source on a per-hectare-of-avocado basis and subsequently converting these values to a per-tonne-of-avocado basis using the average yield per hectare. Further details related to the methodology are provided in later sections as well in the supplementary information.

## Life Cycle Inventory Analysis

This section summarises the Life Cycle Inventory (LCI) analysis for each life cycle stage. Due to the proprietary nature and volume of certain data, specific details are not included. Details of key data required for future studies are provided in the supplementary information. The omission of specific proprietary data, such as fertiliser and pesticide product names and application rates per plantation, is intended to safeguard the intellectual property of relevant enterprises. This precaution is crucial as these enterprises have heavily invested in research and development to optimise avocado production methods, particularly through the utilisation of fertilisers and pesticides tailored to factors like plantation age, yield values, and energy use. Disclosing such information would compromise the competitive edge these enterprises have gained. Main stages are aggregated, with key inventory items highlighted for future studies. For instance, farming involves numerous processes, such as planting and pruning, which are consolidated into singular inventory items. This approach prevents the study from becoming overly burdensome and ensures that it provides meaningful value to the research community.

### Energy

This study examines eight energy sources used in an avocado’s life cycle, including electricity from various national grids and different fuels. South Africa’s electricity, predominantly coal-based, is supplemented by renewables. However, its environmental impact is higher than most countries, particularly those in Europe. The study assumes South African liquid fuel and engines generally conform to Euro V diesel requirements. Energy generation factors and fuel indicator factors are detailed in Table S1 in the supplementary information (CER 2019; EEA 2019; Eskom 2021; UK DoBEIS 2022b).

### Farming

The primary farming stage covers activities from seed preparation to harvesting, involving tasks like planting, fertilisation, pesticide use, and water provision. Notable inputs include large-scale liquid fuel (diesel, petrol, and aviation fuel) and electricity for water pumping. Fertilisers are categorised into Nitrate or Compound-based types in line with protecting proprietary information. Pesticides and lime are also considered and categorised in a similar manner (Audsley et al. 2009; Gaidajis and Kakanis 2021). The indicator factors for the above-mentioned fertilisers and pesticides are shown in Table S5 and Table S6 of the supplementary information.

This study examines the carbon footprint mitigation achieved through composting green waste and solid manure, as well as the carbon sequestration from preserved natural landscapes such as shrublands and savannas. These practices have a net positive impact on the total carbon footprint of compost (Vergara and Silver 2019; US EPA 2020), and enterprises actively engaging in composting are often awarded carbon credits (Kollah et al. 2014).

Indicator factors for the farming phase are split into three, namely 1) liquid fuel use (diesel, petrol and aviation fuel), 2) fertiliser and pesticides, and 3) electricity (required to maintain pump systems, lighting, security, etc. but excludes the packhouse).

#### Liquid fuel use

Various internal combustion engines, including vehicles, helicopters, equipment, trucks, pumps, and generators, are crucial for avocado farm maintenance during the farming phase. Diesel, petrol, and aviation fuel consumption relevant to avocados are measured as 726.65, 14.33, and 8.01 litres(L)/hectare/year, respectively. Utilising Table S1 factors and calorific values for diesel fuel, calculated indicator factors are summarised in Table 1, considering avocado production per hectare (detailed in Table S3).

**Table 1:** Liquid fuel use indicator factors for a typical avocado farm per tonne

Indicator	Unit	Diesel Use	Petrol Use	Aviation Fuel Use
Usage per tonne avocado	L/tonne	81.37	1.60	0.90
Energy	MJ/tonne	2 894.41	52.07	39.36
Carbon dioxide Emissions	kg CO_2_e/tonne	217.96	3.75	2.85
DQI Score		95%		92%
DQI Reference		III, IV, VI		III, IV, V

#### Fertiliser and Pesticides

Fertilisers and pesticides considered during the farming phase consist of Nitrate or Compound-based fertilisers, and pesticides aimed at controlling pathogens, insects, and unwanted vegetation are categorised as either fungicides, insecticides or herbicides. Lime indicators (Blaauw and Maina 2021) are also employed. Utilising the indicator factors listed in Table S5 and Table S6, together with the assumptions listed in Table S3, the indicator factors for fertilisers and pesticides are calculated, as shown in Table 2.

**Table 2:** Fertiliser and pesticide use indicator factors for a typical avocado farm per tonne

Indicator	Unit	Fertiliser^a^	Fungicide	Insecticide	Herbicide	Lime
Usage per tonne avocado	kg/tonne	6.81	0.88	0.04	0.02	6.55
Energy	MJ/tonne	152.29	372.24	9.59	8.47	1.59
Carbon dioxide Emissions	kg CO_2_e/tonne	5.48	25.68	0.66	0.59	0.31
DQI Score		93%	90%			90%
DQI Reference		I, VI, IX	I, VI, X			V, XI

^a^Including Nitrate and Compound-based (phosphate and potassium) fertilisers

#### Farm electricity usage

Farm electricity usage accounted for during the farming phase is a cumulative annual average for eight mature avocado farms, including energy use for irrigation, lighting, security, maintenance, etc. An average of 319 kWh/tonne is calculated. Utilising the indicator factors listed in Table S1, the indicator factors for farm electricity usage are calculated, shown in Table 3.

**Table 3:** Farm electricity use indicator factors per tonne avocados

Indicator	Unit	Farm
Energy	MJ/tonne	1148.4
Carbon dioxide Emissions	kg CO_2_e/tonne	330.74
DQI Score		100%
DQI Reference		I, VI

### Packhouse and Packaging

The packhouse phase involves avocado washing, processing, sorting, on-site refrigeration, and packaging with cardboard boxes. To streamline, avocado washing, processing, sorting, and on-site refrigeration are categorised as ‘Packhouse,’ while the environmental impact of cardboard packaging, a significant contributor, is presented separately. Using the same methodology, indicator factors for packhouse and cardboard packaging are calculated as shown in Table 4.

**Table 4:** Packhouse and cardboard packaging indicator factors for a typical avocado farm

Indicator	Unit	Packhouse	Cardboard
Usage per tonne	kWh/tonne	38.21	–
Usage per tonne	kg/tonne	–	78.03
Energy	MJ/tonne	137.54	1 711.00
Carbon dioxide Emissions	kg CO_2_e/tonne	39.6	64.06
DQI Score		96%	87%
DQI Reference		I, V	I, VI, XII, XIII

### Transport to Cape Town and Loading on Ship

For long-distance transportation to Cape Town, indicator factors are determined, similar to those in the previously outlined process. Notably, avocados undergo refrigeration using reefer containers, drawing power from the truck’s auxiliary supply at an assumed 90% efficiency (Fitzgerald et al. 2011). The 1,810 km journey from Tzaneen to the Port of Cape Town takes 20 hours, with a 2% inefficiency considered for loading and unloading delays. Based on a 300,000 km fuel consumption road test (Reincke Logistiek 2022) by the logistics company hauling this produce, the typical truck hauling 24 tonnes of avocados requires 0.014 L/tonne-km. Utilising the factors from Table S1, the calculated indicator factors for transporting one tonne from Tzaneen to Cape Town are presented in Table 5, reflecting business-as-usual transportation (Note: Table 5 depicts results under standard transportation conditions).

**Table 5:** Indicator factors to transport one-tonne product from Tzaneen to Cape Town

Indicator	Unit	Leg 1 (Long distance haul)	Leg 1 (Refrigeration)	Leg 1 (Total)
Energy	MJ/tonne	919.40	8.91	928.31
Carbon dioxide Emissions	kg CO_2_e/tonne	69.23	0.67	69.91
DQI Score		94%	89%	93%
DQI Reference		III, IV, VI, VIII, XXII	III, IV, VIII, XIV	III, IV, VI, VIII, XIV

### Transport by Ship to Rotterdam

Container ships, measured by TEU capacity, play a crucial role in this study, with the specific vessel having a capacity of 9162 TEUs. Verified smAvo data, supplemented by FleetMon live tracking, reveals the ship’s average speed at 18.75 knots over a 12,500 km journey (Cape Town to Rotterdam). Fuel consumption is estimated using Nottenboom and Carriou’s (2009) data, applying 160 tonnes of marine fuel per day with inefficiencies. Energy for reefer container cooling is similarly assessed. Reefer delays of two days in Cape Town and three days in Rotterdam are factored in, considering electricity from national grids. A 1% inefficiency, reflecting loading and unloading, is incorporated based on stakeholder input. Utilising Table S1 factors, the calculated indicator factors for transporting one tonne from Cape Town to Rotterdam are detailed in Table 6.

**Table 6:** Indicator factors to transport one-tonne product from Cape Town to Rotterdam

Indicator	Unit	Leg 2 (Long distance shipping)	Leg 2 (Refrigeration)	Leg 2 (Total)
Energy	MJ/tonne	750.69	375.09	1 125.78
Carbon dioxide Emissions	kg CO_2_e/tonne	59.80	35.05	94.85
DQI Score		87%	93%	90%
DQI Reference		III, IV, VIII, XV	I-IV, VIII, XIV	I-IV, VIII, XIV, XV

### Ripening chamber

Fresh climatic fruits, like avocados, are commonly imported green and ripened artificially using ethylene in dedicated chambers. This study, acknowledging the perceived carbon intensity of this ripening process, contrasts it with an alternative where avocados are sold green, and consumers facilitate natural ripening domestically. While ripening chamber specifics are often proprietary, this study assumes a conservative model—a 56 m^3^ chamber with a 160,000 avocado capacity, operating at 16–18 °C and 85% humidity for ~28 hours. The estimated energy requirement for the chamber is 3400 Btu/hr. Catalytic generation is assumed as the preferred ethylene addition method, with an electrical generator requiring 20,000 Btu for the 28 h period. According to Worrell et al. (2000), ethylene production demands 26 MJ/kg, with 3 litres over the 28-hour period. Two ripening scenarios, Rotterdam and Paris, are explored, considering power from the Dutch and French national grids, respectively. Table 7 consolidates indicator factors, combining data from Table S1, to illustrate the environmental impact of ripening one tonne of product in each scenario.

**Table 7:** Indicator factors for ripening one tonne of avocados

Indicator	Unit	Ripening – Rotterdam	Ripening - France
Energy	MJ/tonne	201.05	
Carbon dioxide Emissions	kg CO_2_e/tonne	21.71	3.13
DQI Score		79%	
DQI Reference		II, XVI, XVII	

### Delivery to retail

Transportation from the distribution warehouse to retail is crucial in the final stage of the avocado life cycle. Three scenarios are analysed for their indicator factors. Scenario 1 involves local delivery to a Rotterdam retailer (50 km assumed distance), Scenario 2 envisions a 450 km journey to Paris, France, and Scenario 3 anticipates a 700 km trip to Nottingham, England. It is assumed the truck uses the Eurotunnel for the English Channel crossing. Refrigerated containers are employed throughout each scenario. Applying data from Table S1 and specified fuel usage rates, Table 8 outlines the calculated indicator factors for transporting one tonne of avocados in each scenario.

**Table 8:** Indicator factors for transportation and refrigeration of one tonne of avocados from the Port at Rotterdam to various destinations

Indicator	Unit	Scenario 1 (Rotterdam - Local)	Scenario 2 (Paris, France)	Scenario 3 (Nottingham, England)
Energy	MJ/tonne	25.35	226.77	354.81
Carbon dioxide Emissions	kg CO_2_e/tonne	1.91	17.08	26.61
DQI Score		86%		83%
DQI Reference		VI, XIV		VI, XIV, XVIII

### Carbon Sequestration

Carbon sequestration, a pivotal aspect in climate change mitigation, plays a central role in this LCA. The process involves capturing and storing greenhouse gas emissions, with enterprises actively participating in large-scale carbon sequestration to reduce their net carbon footprint and potentially earn carbon credits.

#### Methodologies for Carbon Sequestration Calculation

The study focuses on an agricultural enterprise engaged in carbon sequestration, aligning with the European Commission’s criteria that only credits sequestration beyond normal activities. The farm, featuring expansive pine and eucalyptus plantations, preserved shrubland, active composting initiatives, avocado trees, onion and tomato plantations, among others, serves as a significant carbon sink. Notably, plantations used for income under European Law’s business-as-usual scenario are excluded from consideration.

To quantify carbon sequestration, a comprehensive approach is utilised, drawing data from both the enterprise and relevant literature. The methodologies for calculating carbon sequestration per hectare per year and per tonne of avocado product are outlined in Table 9.

**Table 9:** Carbon Sequestration potential for one tonne of product

Carbon Sequestration Sources (DQI Reference)	Carbon sequestration per hectare per year (tonne CO_2_e/ha/year)	Carbon Sequestration per tonne product (tonne CO_2_e/tonne avocado)
Shrubland (Luo et al. 2006)	1.90	0.012
Preserved Savannah (Zhou et al. 2022)	0.35	0.061
Compost (manure and green waste) (Vergara and Silver 2019)	5.81	0.449
Total Carbon Sequestration Potential	8.03	0.522
DQI Score^a^	89%	
DQI Reference	V, XIX–XXI	

^a^Includes DQI Reference VI for hectare data

#### Variability in Carbon Sequestration Estimates

It is imperative to acknowledge the inherent variability in estimating carbon sequestration. Factors such as soil types, climate variations, and agricultural practices contribute to fluctuations in sequestration rates. Recognising this, the study incorporates a conservative approach, considering potential uncertainties in calculations. Further clarification of uncertainties are provided in later sections and in the supplementary information.

## Life Cycle Impact Assessment

**Fig. 2:**
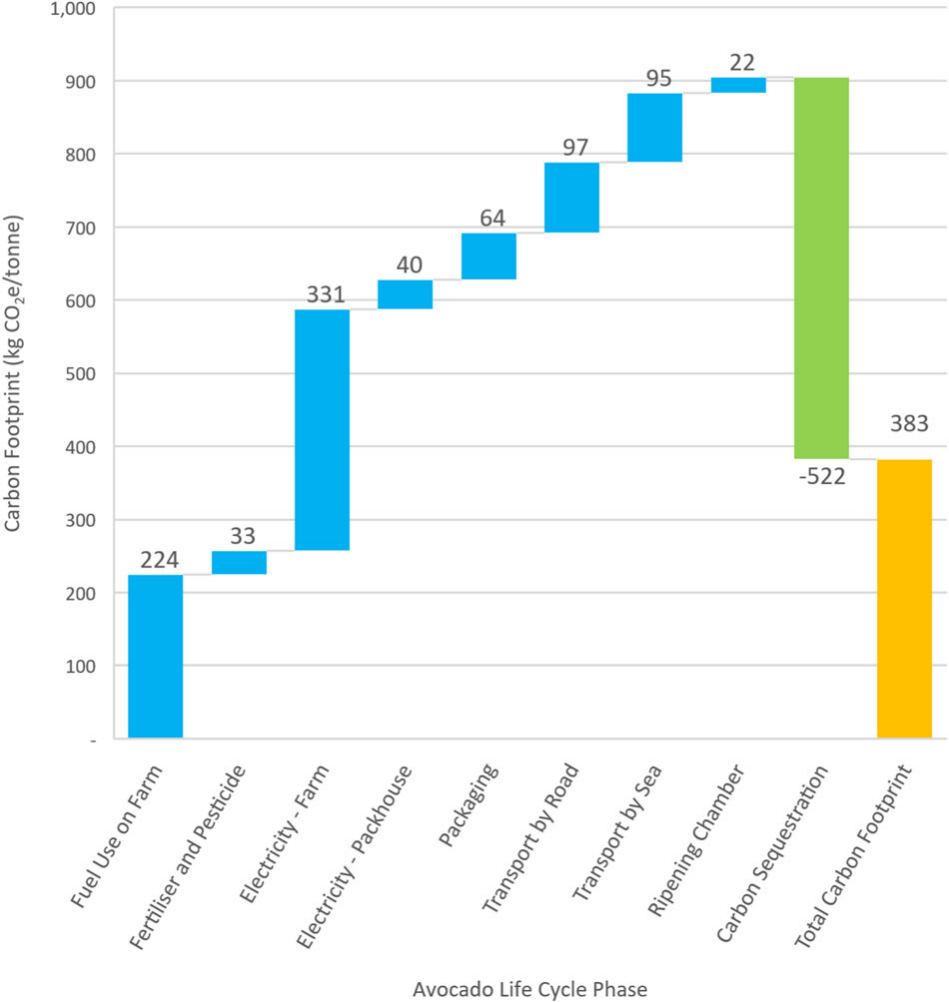
Avocado life cycle assessment results

Utilising the results of the LCI for the various life cycle phases previously described of a typical avocado grown in South Africa and enjoyed in Europe, the Life Cycle Impact Assessment (LCIA) may be calculated for the base year of 2022 and a functional unit of one-tonne product. Figure 2 illustrates LCIA results, with a total carbon input of 904.85 kg CO_2_e/tonne. Mitigating this, 521.88 kg CO_2_e/tonne is offset, resulting in a net carbon footprint of 382.97 kg CO_2_e/tonne or 57.45 g CO_2_e/avocado grown in South Africa and sold in Europe.

To contextualise carbon sequestration activities within the framework of conventional avocado farming in South Africa, it is essential to distinguish between business-as-usual practices and those that go beyond standard agricultural operations. In conventional avocado farming, typical practices may not inherently include extensive carbon sequestration efforts. Business-as-usual activities largely focus on the cultivation and harvesting of avocados, employing standard agricultural practices to maximise yield and efficiency. These practices might involve basic soil management, irrigation, pest control, and fertilisation, without necessarily prioritising carbon sequestration. However, some farms in South Africa might adopt additional sustainable practices that incidentally contribute to carbon sequestration, such as integrating trees within avocado plantations (agroforestry), enhancing soil health and organic matter through cover cropping and mulching, and reducing soil disturbance with minimal tillage.

This study, however, focuses on an agricultural enterprise engaged in carbon sequestration beyond these conventional activities, aligning with the European Commission’s criteria that only credits sequestration beyond normal activities. This farm employs a range of advanced practices specifically designed to enhance carbon sequestration. Expansive pine and eucalyptus plantations, as well as avocado, onion, and tomato plantations, even though sequestering carbon, do not count towards carbon sequestration potential as they form part of the business model and are used for income. In contrast, activities such as active composting initiatives, maintained shrubland, and preserved savannah go beyond the business requirements, thus counting towards the carbon sequestration potential. Such enhanced activities are not yet widespread or considered common practice in the area, representing a significant step beyond traditional farming methods. Therefore, the quantified carbon sequestration in this study reflects deliberate and additional efforts undertaken by the farm to exceed normal operational activities, contributing substantially to climate change mitigation.

In addition, the study identifies energy-intensive hotspots, suggesting optimisation opportunities including: adopting renewable energy, exploring alternative packaging, delivering unripe avocados, and minimising stages in the logistics chain. The subsequent section conducts a sensitivity analysis to investigate these identified hotspots further.

### Sensitivity Analysis

This section conducts a sensitivity analysis of the presented life cycle results to pinpoint significant carbon footprint reduction opportunities for imported avocados. It is divided into two segments: 1) assessing the carbon footprint reduction achieved through ongoing carbon-saving measures actively pursued by stakeholders, and 2) exploring additional measures for stakeholders to attain Net Zero, surpassing current active initiatives. The aim is to guide optimisation efforts and highlight innovative interventions or process changes deserving further investigation by relevant enterprises. Detailed information of the sensitivity information is provided in the supplementary information.

The study evaluates several strategies to reduce the environmental footprint of avocado production and distribution. Firstly, transitioning to renewable energy sources, such as solar power, for farm and packhouse operations offers substantial emissions reductions. Results show that large-scale use of renewable energy can eliminate up to 368.21 kg CO_2_e/tonne from the avocado’s life cycle carbon footprint, equivalent to a 96.15% reduction. Solar panel quantity and land requirements for 100% renewable energy operation are also assessed, suggesting that 1.1 to 1.4 hectares of solar panels can power the total 1025 hectares of avocado plantations (inclusive of mature and developing plantations). In this analysis, the potential impacts of land-use change associated with renewable energy infrastructure implementation were initially explored. However, it was found that the proposed changes would have minimal environmental implications. Specifically, converting a small portion of the currently preserved savannah land mass (1.1 to 1.4 hectares out of 6000 hectares) for solar panel installation represents only a 0.02% alteration in land use. This negligible change falls well within industry-accepted standard error ranges for assessments of this nature and has an insignificant effect on carbon sequestration potential. While accounting for upstream emissions related to this land-use change through the selection of indicator factors for solar energy, detailed in the supplementary information, it was ultimately decided that further elaboration on this aspect would not significantly enhance the value of this paper.

Another aspect considered is the impact of delivering avocados unripened compared to artificially ripened. Selling avocados unripened, allowing natural ripening by consumers, can reduce total carbon footprint emissions by 5.67%, equivalent to eliminating up to 21.71 kg CO_2_e/tonne of avocados. Furthermore, alternatives to cardboard packaging, such as reusable plastic containers, are explored. Shifting from single-use cardboard to reusable plastic containers could decrease the packaging life-cycle phase emissions by 58.11 kg CO_2_e/tonne (15.17%), highlighting a significant reduction in environmental impact.

The study also addresses wastage reduction, a critical aspect affecting economic, environmental, and food security metrics. Total waste for avocado farms is calculated as 590 kg (6.73%) avocados per hectare per year and 30 kg (3%) per tonne transported to Europe. This equates to a combined 848.45 kg (9.85%) avocados per hectare per year lost to spoil, resulting in a carbon footprint of 3.48 kg CO_2_e/tonne avocado (0.91%). Considering the carbon footprint linked with decreased wastage is essentially akin to boosting yield per hectare, a topic explored in the following section on uncertainty analyses. It’s worth noting that a 9.85% rise in yield (factoring in the elimination of wastage) merely results in a perceived marginal decrease of 0.91% in carbon emissions. This occurs because, with increasing yield due to zero wastage, less carbon sequestration potential is allocated to each tonnage per hectare, counterbalancing the emission reduction when examining the avocado’s holistic life cycle.

**Fig. 3:**
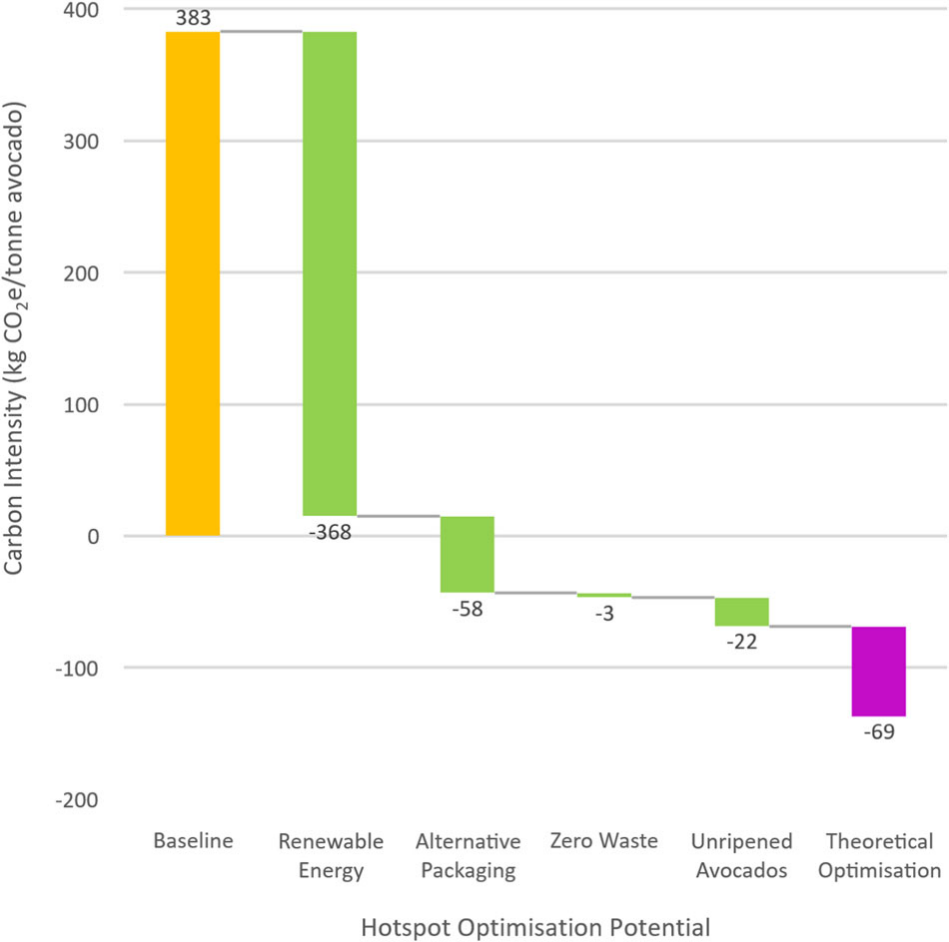
Optimisation potential for the avocado life cycle

Incorporating these carbon footprint mitigation measures could lead to a theoretical 117.90% reduction (382.97 kg CO_2_e/tonne to −68.54 kg CO_2_e/tonne) in avocados’ total life cycle impact, potentially achieving ambitions of Net Zero emissions. The sensitivity analysis results are presented in Fig. 3 and detailed in the supplementary information.

### Uncertainty Analysis

The uncertainty analysis methodology comprises several key steps to comprehensively assess and address uncertainty within the study’s framework. Firstly, the exploration of scenario uncertainty involves considering a range of hypothetical scenarios that capture variations in critical factors across different phases of the avocado’s life cycle. Additionally, Monte Carlo simulations are employed for the various scenarios to derive upper and lower quartile values to illustrate uncertainty. These simulations generate multiple iterations, each representing a possible outcome based on the probability distributions of input parameters.

Furthermore, a critical aspect of the methodology involves the assessment of data quality. This step addresses concerns regarding the reliability of the dataset by acquiring updated indicator factor data relevant to the products and processes under scrutiny. By refining the information with improved dataset integration into Monte Carlo simulations, uncertainties stemming from data quality are accounted for. The resulting upper and lower quartile values reveal the extent of uncertainty attributable to data reliability.

Finally, the analysis and comparison stage systematically evaluate uncertainty ranges represented by upper and lower quartile values across all life cycle phases. Aggregating these uncertainty ranges enables the overall impact of uncertainty on assessment results to be gauged.

Table S25 in the Supplementary Information details the uncertainty assessment methods and parameters considered across all avocado production and distribution life cycle phases, summarised here in Table 10.

**Table 10:** Uncertainty assessment methods for various life cycle phases in agricultural production

Life Cycle Phase	Uncertainty Method	Uncertainty Parameters
Liquid fuel use on Farm	Scenario Analysis	Variability in fuel consumption volume
Fertiliser and Pesticide	Data Quality Assessment	Reliability of indicator factors for fertilisers and pesticides
Electricity - Farm	Scenario Analysis	Variation in energy consumption per hectare
Packaging	Data Quality Assessment	Accuracy of indicator factors for cardboard boxes
Transport by Road	Data Quality Assessment	Precision of truck fuel usage per kilometer
Transport by Sea	Scenario Analysis	Fluctuations in marine fuel usage, trip duration, port waiting times, ship size, and ship speed
Ripening Chamber	Scenario Analysis	Seasonal variation in energy usage for cooling
Carbon Sequestration	Data Quality Assessment	Dependability of indicator factors for carbon sequestration potential
Yield	Scenario Analysis	Changes in yield per hectare per year

In the liquid fuel use on the farm phase, scenario analysis is employed to account for variability in fuel consumption volume, allowing for the exploration of different fuel consumption scenarios. For fertiliser and pesticide usage, a data quality assessment approach is adopted, focusing on the reliability of indicator factors to ensure accuracy in assessing environmental impacts associated with their use. Similarly, electricity consumption on the farm undergoes scenario analysis to capture variations in energy consumption per hectare, addressing uncertainties arising from fluctuating energy demands.

Concerning packaging materials, a data quality assessment is conducted to evaluate the accuracy of indicator factors for cardboard boxes, ensuring the reliability of environmental impact assessments related to packaging. For road transport, data quality assessment is utilised to assess the precision of truck fuel usage per kilometre, enhancing the reliability of estimates for fuel-related emissions during transportation. In contrast, uncertainty in sea transport is addressed through scenario analysis, considering fluctuations in marine fuel usage, trip duration, port waiting times, ship size, and speed, reflecting the variability inherent in maritime transportation.

**Fig. 4:**
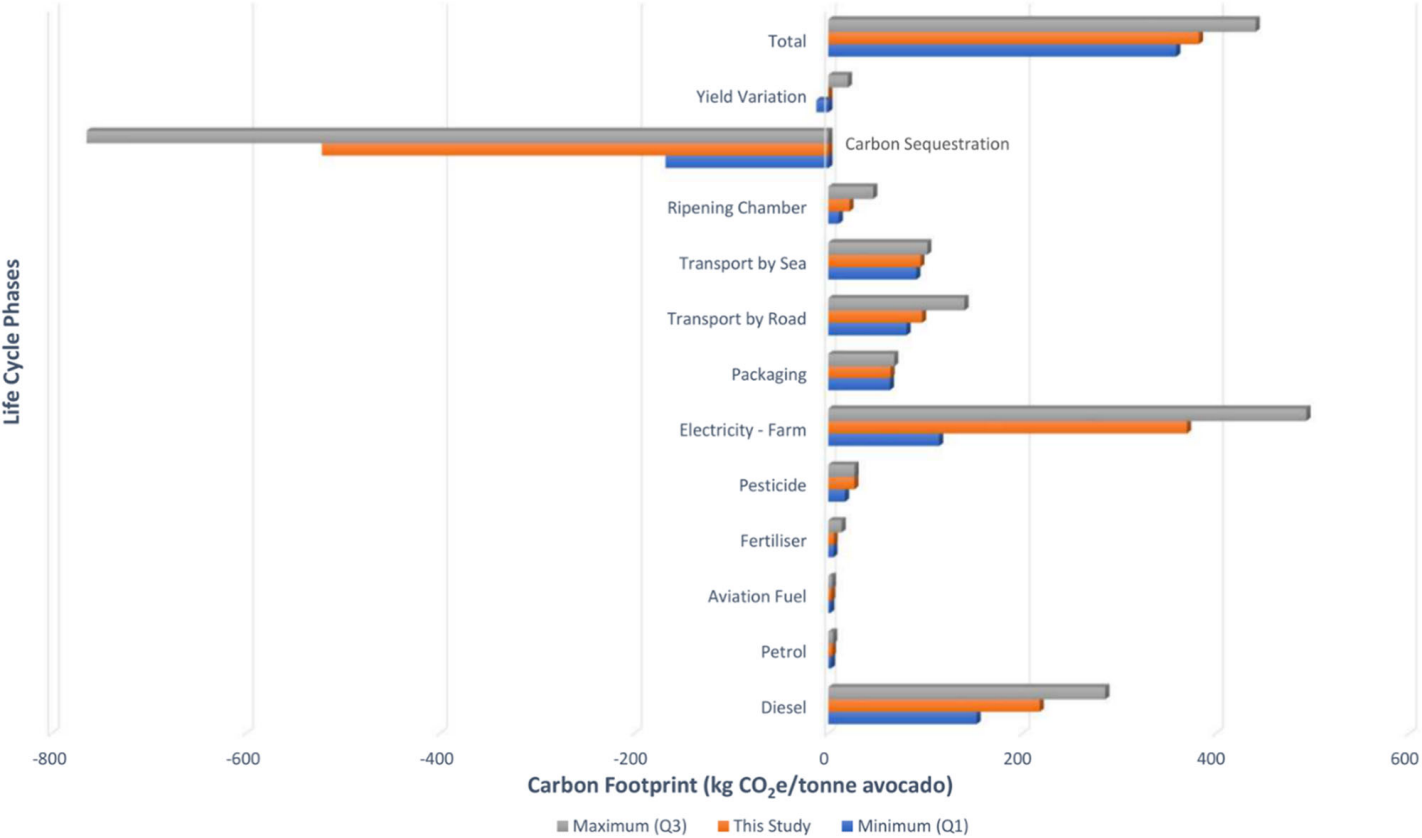
Uncertainty Analysis Results for one-tonne avocado

In the ripening chamber phase, scenario analysis is employed to account for seasonal variation in energy usage for cooling, acknowledging the dynamic nature of energy consumption patterns. Additionally, a data quality assessment approach is applied to assess the dependability of indicator factors for carbon sequestration potential, ensuring the accuracy of estimates related to carbon storage. Lastly, yield variations are addressed through scenario analysis, allowing for the exploration of changes in yield per hectare per year, capturing uncertainties associated with agricultural productivity. The uncertainty analysis findings are presented in Fig. 4, with additional details available in the supplementary information. Notably, uncertainty exists, particularly regarding farm fuel and energy usage, as well as carbon sequestration potential in shrublands, savannahs, and composting. However, the aggregation of positive and negative impacts reveals a moderated total uncertainty for one tonne of avocado, ranging from −23.22 to +58.69 kg CO_2_e (equivalent to −6.1 to +15.3%).

### Projected Climate Change Impacts

This study delves into the prospective implications of climate change on the sustainability of avocado farming in South Africa. Climate change, characterised by incremental shifts in temperature and moisture conditions owing to heightened concentrations of atmospheric CO_2_e emissions, brings with it an augmented frequency and intensity of extreme weather events, such as storms, floods, hot days, and prolonged droughts. These events amplify short-term repercussions on society, the environment, logistical supply chains, and enduring climatic changes.

Employing an updated Thornthwaite Moisture Index (TMI) specific to South Africa (Blaauw et al. 2022), as depicted in Figure S7 in the Supplementary Information, which gauges soil and climate aridity or humidity and is widely utilised in agriculture, the study anticipates both gradual micro-climatic alterations and an uptick in extreme weather events. These changes are projected for the farm under consideration, situated in the Greater Tzaneen region. The region’s micro-climate is forecasted to transition into a more humid tropical climate, accompanied by an increase in very hot days and a heightened flood risk, agricultural vulnerabilities, and water supply vulnerability (Engelbrecht et al. 2019).

These environmental stressors are expected to impact various facets of plant life, influencing productivity, chemistry, defences, nutritional quality, palatability, and digestibility (Xie et al. 2019). The gradual warming and increased humidity are projected to regulate insect physiology and metabolism, potentially intensifying crop damage due to increased calorific intake and higher growth rates among herbivore insects (Deutsch et al. 2018). Notably, the reduction in projected drought tendencies is anticipated to benefit avocado cultivation, which necessitates a continuous source of moisture.

**Fig. 5:**
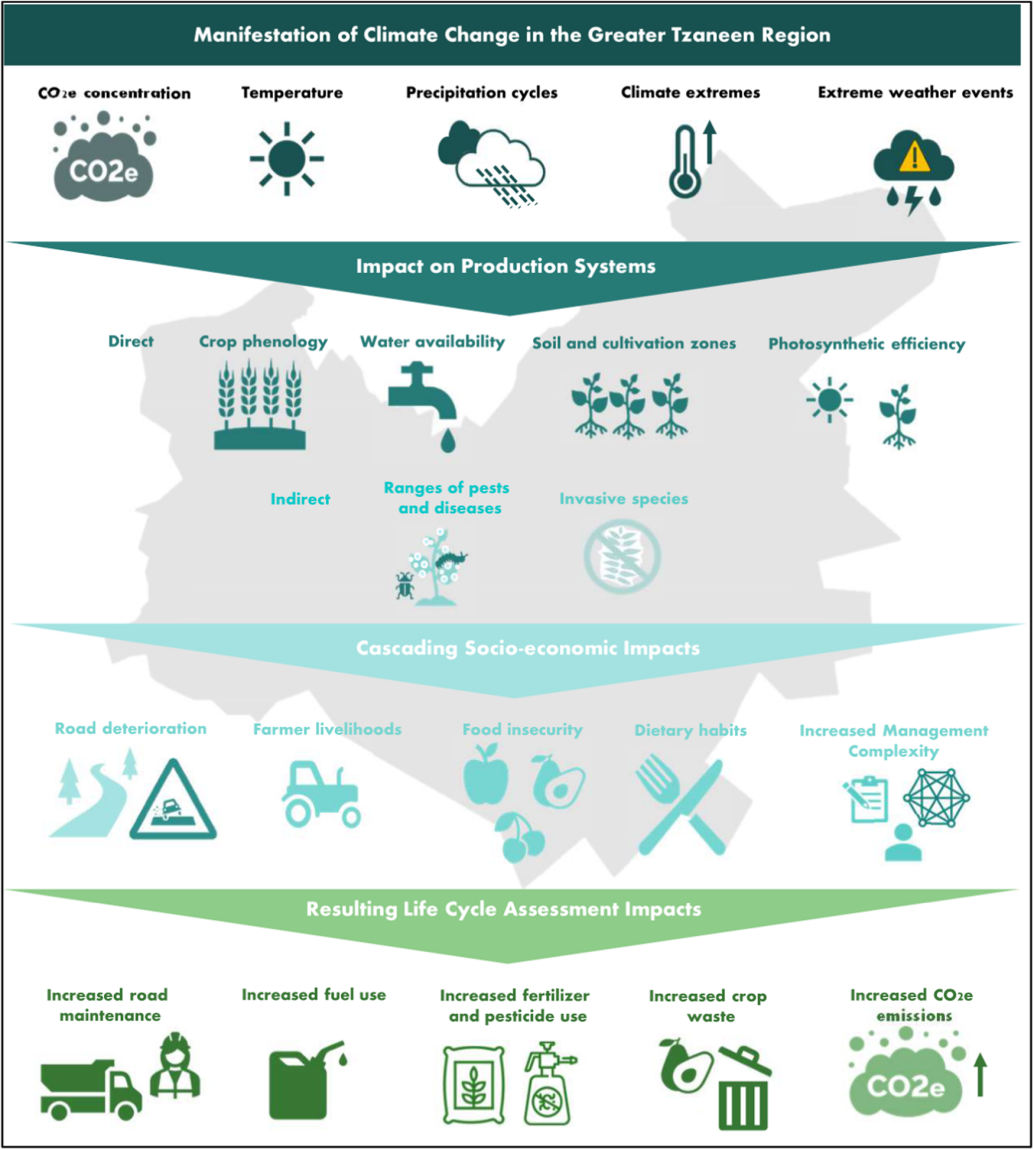
Impact of climate change on the Greater Tzaneen micro-climate agricultural landscape

The study also extends its focus beyond agriculture, revealing that road infrastructure performance in the Greater Tzaneen region will deteriorate, contrary to the general trend in most of South Africa (Blaauw et al. 2022). This deterioration demands enhanced maintenance, raises the risk of produce damage, and increases fuel consumption, contributing to elevated emissions. Avocado plantations, predominantly situated in micro-climates becoming wetter and warmer, may face more significant risks associated with climate change compared to other agricultural sectors. Figure 5 illustrates the manifestation of climate change for the Greater Tzaneen micro-climate, its impacts on production systems, cascading socio-economic impacts and resulting LCA impacts.

In light of these findings, the study recommends that avocado farmers assess the potential impact of climate change on their plantations, evaluate the resilience of their systems, and implement proactive mitigation measures. Additionally, it advocates for local and national governmental support to develop the resilience of avocado farmers in South Africa, recognising the heightened vulnerability of the avocado industry compared to other agricultural sectors in the face of changing climate conditions.

## Discussion

The LCIA results, derived from the LCI of avocados grown in South Africa and consumed in Europe developed in this study, provide valuable insights into the environmental footprint of this supply chain. With a functional unit of one tonne of product, the LCIA for the 2022 base year reveals a total carbon input of 904.85 kg CO_2_e per tonne of avocados without any carbon sequestration applied. However, it is noteworthy that 521.88 kg CO_2_e per tonne is offset through sequestration and composting, resulting in a net carbon footprint of 382.97 kg CO_2_e per tonne or ~57.45 g CO_2_e per avocado.

**Fig. 6:**
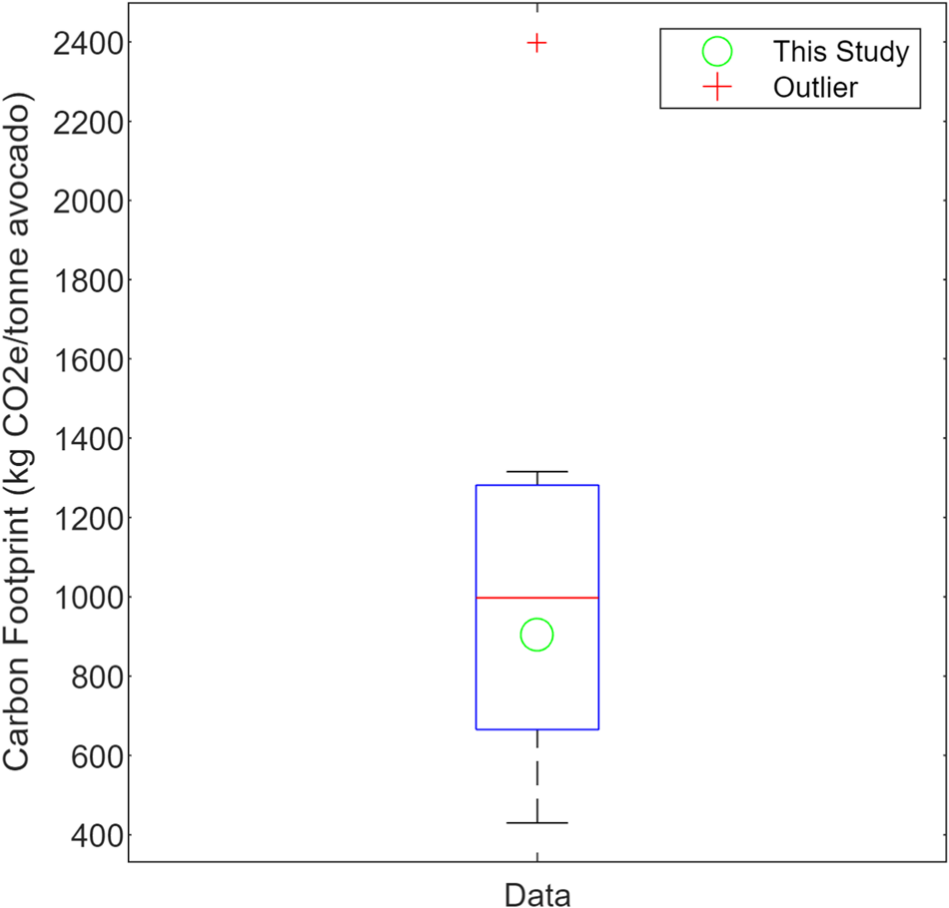
Comparison against other studies (Audsley et al. 2009; Stoessel et al. 2012; Bell et al. 2018; Frankowska et al. 2019; NL-NIPHE 2021; Esteve-Llorens et al. 2022; Majumdar and McLaren 2023)

Research on the life cycle impact of the avocado industry is scarce and often fraught with challenges due to regional variations. Du Plessis et al. (2022) provide insights into LCA results for South African avocados, complemented by studies from Audsley et al. (2009), Stoessel et al. (2012), Frankowska et al. (2019), Esteve-Llorens et al. (2022), Majumdar and McLaren (2023) and LCAs on stone fruits (Martin-Gorriz et al. 2020), as well as shipping values (Mærsk 2021; UK DoBEIS 2022b), among others. However, existing literature lacks consistency in methodology and often relies on defaults and assumptions, potentially resulting in overestimated impacts and disregarding factors like carbon sequestration and the influence of the ripening chamber life cycle, which this study is the first to investigate. Despite these challenges, Table S32 summarises the available literature for comparison. When compared to these studies sharing similar system boundaries (farm-to-fork excluding carbon sequestration), it is observed that this study’s findings fall within comparable ranges, as demonstrated in Fig. 6. Figure 6 also serves to highlight another critical facet of LCA studies, emphasising their constraint resulting from insufficiently large databases for statistical analyses. In conjunction with prior publications, the current study lays the foundation for a database concerning avocado carbon footprints. Furthermore, the findings of this investigation augment the precision of future estimations regarding the carbon footprints of avocados.

Notably, the farming life cycle phase, specifically fuel use (petrol, diesel and aviation fuel) as well as electricity consumption (lighting, security, irrigation, etc.) are consistently leading contributors to the avocado life cycle impacts across studies, in line with the findings of this study where the farming phase accounts for over 60% of emissions generated. The relevant enterprises should further investigate measures to mitigate these impacts. This study has, however, investigated potential mitigation measures through sensitivity analyses.

It is important to understand that while there are various approaches to conducting sensitivity analyses for optimisation, this study has chosen to concentrate on areas actively pursued individually. This decision was driven by the availability of reliable data suggesting feasibility while excluding mitigation options not currently under investigation or consideration and are deemed unlikely to be pursued in the future. Sensitivity analyses show that the active measures pursued by the relevant enterprises have the potential to meet aspirations for Net Zero, reducing the baseline emissions from 382.97 kg CO_2_e/tonne to a theoretical −68.54 kg CO_2_e/tonne (117.9% decrease). Similar studies (Stoessel et al. 2012) have reported comparable reduction potential.

This study has assessed various scenarios and data quality uncertainties across all life cycle phases, utilising the widely accepted Monte Carlo simulations approach. This method helps establish upper and lower bounds of variation to account for uncertainty. The findings suggest that the most significant uncertainties arise from the farm’s liquid fuel, electricity consumption, and carbon sequestration. The maturity of the plantation often influences the level of uncertainty in farm activities and resource usage per hectare. Less mature plantations typically require significantly more resources for establishment than well-established ones, thus impacting the variability of results. It is, however, essential to highlight that the impact of yield (influenced by plantation maturity) on outcomes is relatively insignificant due to the dependence of carbon sequestration on yield per hectare. As yield decreases, more carbon sequestration is attributed to each tonnage per hectare, offsetting increased emissions.

Conversely, with increasing yields, less carbon sequestration is attributed to each tonnage per hectare, thereby aggravating expected decreases in carbon intensity associated with higher yields. A comparable occurrence arises in the assessment of uncertainty concerning carbon sequestration. This study utilised indicator factor data from secondary published sources (Luo et al. 2006; Vergara and Silver 2019; Zhou et al. 2022) that closely matched the study area for the LCIA. However, when incorporating a broader database of published indicator factors (detailed in the supplementary information) for uncertainty analysis, it becomes evident that this study has greatly underestimated the carbon sink potential of savannahs and shrublands and has employed more cautious values regarding composting carbon sequestration potential. Likewise, through aggregation and integration of results, they counterbalance each other, yielding uncertainty ranges for sequestration potential closely in line with the estimates presented in the LCIA, with total combined uncertainty ranging from −23.22 to +58.69 kg CO_2_e/tonne avocado (equivalent to −6.1 to +15.3%). Similar ranges for variations in LCIA results for avocados are reported by Bartl et al. (2012), Bell et al. (2018), and Esteve-Llorens et al. (2022). Few studies (Graefe et al. 2013; Bell et al. 2018) consider climate change’s influence and its uncertainty.

The study investigates the potential consequences of climate change on avocado farming sustainability in South Africa. A rise in extreme weather events and alterations in micro-climatic conditions are anticipated, particularly in the Greater Tzaneen region, where the farm is located. These changes are expected to influence various aspects of avocado cultivation, including productivity, pest pressure, and water availability. Additionally, the study highlights the broader impacts of climate change on infrastructure, such as road networks, which could exacerbate challenges in transportation and logistics for avocado farmers. Given these projections, it is recommended that avocado growers assess their vulnerability to climate risks and implement adaptive measures. It also underscores the importance of governmental support in fostering resilience within the avocado Industry amidst changing environmental conditions.

### Study Limitations

While this study provides valuable insights into the LCA results of South African avocado production, it is important to acknowledge the inherent limitations that may affect the interpretation and generalisability of the findings.

One of the primary limitations is the reliance on available data. In sectors where information is proprietary, this reliance may introduce additional uncertainties and necessitate certain assumptions. This is a common challenge in research and underscores the importance of transparency about data sources and methodologies. The geographical focus of the study is another limitation. Concentrating on the Greater Tzaneen region means the findings may not broadly apply to regions with different agricultural practices. Agriculture is diverse, and practices can vary widely even within the same country.

Temporal constraints also play a role. The study reflects the state of knowledge up to a certain point in time and may not capture recent developments in the field. This is particularly relevant given the dynamic nature of agriculture and the ongoing uncertainties in climate change projections. Finally, delineating the study’s scope and the methodological choices can introduce potential variations. Each study must define its boundaries, and these choices can affect the results.

Despite these limitations, the study makes a significant contribution by establishing a foundational understanding of the LCA of South African avocado production. It also highlights the need for ongoing research and adaptability in response to shifts in the industry. As with all research, the findings of this study should be interpreted in the context of its limitations.

### Future Research

Future avocado research holds the potential to mitigate carbon emissions through various avenues including efficient irrigation techniques, quantification of social emissions, waste reduction strategies, exploration of alternative farming practices, analysis of transportation methods, and examination of consumer behavior patterns. By delving into these areas, researchers can gain a more comprehensive understanding of avocado’s environmental impacts and develop effective strategies to address them, thus informing policy decisions, shaping industry practices, and empowering consumers to make more sustainable choices.

## Conclusions

This study presents a comprehensive LCA of the avocado supply chain, considering various scenarios and optimisation initiatives. While the findings are specific to South African avocados exported to Europe, the extensive analysis, real-time data from an 18-month campaign, exploration of mitigation measures and uncertainty analyses, and consideration of climate change contribute significantly to understanding the impact of avocado farming and exporting on life cycle emissions.

Climate change impacts can vary, and while some may be beneficial, it is anticipated that the cumulative effects will be negative in the micro-climatic regions where most South African avocado plantations are located. Therefore, it is crucial to develop and implement proactive mitigation strategies, as discussed in this paper, to enhance the resilience of these plantations to projected future impacts.

### Policy and Implications

The comprehensive LCA of an avocado, as presented in this study, holds substantial implications for policy-making and stakeholder engagement in both South Africa and Europe.

The first implication lies in the realm of policy-making. The granular understanding of the environmental impacts at each stage of the avocado’s life cycle could serve as a foundation for policies aimed at mitigating these impacts. This could manifest in the form of policies promoting sustainable farming practices or optimising transportation logistics to curtail carbon emissions.

The second implication concerns the stakeholders within the avocado industry, including producers, distributors, and retailers. The insights derived from this study could guide their operational decisions, potentially leading to investments in technologies or practices that diminish their environmental footprint. Furthermore, these insights could be leveraged in marketing strategies to cater to the growing segment of environmentally conscious consumers.

The third implication pertains to consumer behaviour. With the rising awareness of the environmental repercussions of food production, consumers are increasingly gravitating towards sustainable options. Detailed information about the life cycle of an avocado could empower consumers to make more informed, environmentally-friendly purchasing decisions.

Lastly, the implications extend to future research. The methodology employed in this study could be replicated for other food products, thereby offering a comprehensive understanding of the environmental impacts of a wide array of foods. This, in turn, could shape future policy decisions and stakeholder behaviour across the broader food industry.

## Supplementary Information

Supplementary Information
