# Supplementary material for: Life Cycle Assessment of an Avocado: Grown in South Africa—Enjoyed in Europe
Source: Environ Manage. 2024 Jun 27;74(5):989–1005. doi: 10.1007/s00267-024-02009-w (PMC11438827; doi:10.1007/s00267-024-02009-w)
Supplement: Supplementary file 1 — Supplementary Information [file 267_2024_2009_MOESM1_ESM.pdf]

Supplementary Information

This supplementary information contains all relevant data utilised in the development of the Life Cycle Inventory for the study "Life Cycle Assessment of an Avocado – Grown in South Africa, Enjoyed in Europe". For the Data Quality Matrix related to this information please see Table S34 at the end of this document.

Contents

Supplementary Information 1

S1. Key Input data and Assumptions 4

S2. Farm Life Cycle Inventory 8

S3. Transportation Life Cycle Inventory 12

S4. Ripening Chamber Life Cycle Inventory 17

S5. Carbon Sequestration Life Cycle Inventory 18

S5.1 Description of Calculation Model Represented in Table S21 19

S6. Sensitivity Analysis 23

S7. Uncertainty Analysis 26

S7.1 Methodology for Uncertainty Assessment 26

S7.1 Example of Data Quality Assessment Uncertainty – Carbon Sequestration 27

S7.2 Example of Scenario Analysis Uncertainty – Farm Energy use per Plantation 32

S7.3 Uncertainty Analysis Results 34

S7.4 Example MATLAB Code to Determine Uncertainty with Monte Carlo Simulations 34

S8. Projected Climate Change Impacts 36

S9. Comparison to Other Studies 37

S10. Data Quality Matrix 38

References: 40

List of Figures

Figure S1: Monte Carlo Results - Box Plot of Uncertainty for Shrubland and Savannah 30
Figure S2: Monte Carlo Results – Probability Density Function of Uncertainty for Shrubland and Savannah 30
Figure S3: Monte Carlo Results – Box Plot of Uncertainty for Composting 31
Figure S4: Monte Carlo Results – Probability Density Function of Uncertainty for Compost 31
Figure S5: Monte Carlo Results - Box Plot of Uncertainty for Shipping Variation 33
Figure S6: Monte Carlo Results – Probability Distribution Function of Uncertainty for Shipping Variation 33
Figure S7: Thornthwaite Moisture Index changes for South Africa (2021–2050) (Blaauw et al., 2022) 36

List of Tables

Table S1: Emissions per Mega Joule Energy 4
Table S2: Fuel Usage Rates 4
Table S3: Assumptions related to Avocado Transportation and Farming 4
Table S4: Fuel Indicator Factors 4
Table S5: Fertiliser Indicator Factors 5
Table S6: Pesticide Indicator Factors 5
Table S7: Farming Enterprise Commodities 6
Table S8: Yield and Energy Use on Farm (various locations excluding packhouses) 7
Table S9: Farm Fuel Usage 8
Table S10: Pesticide Usage 9
Table S11: Electricity usage for the Farm and Processing Packhouse 10
Table S12: Packaging Energy and Emissions Usage 11
Table S13: Leg 1 Fuel Usage and Emissions 12
Table S14: Leg 2 Fuel Usage and Emissions 13
Table S15: Leg 2 Refrigeration Fuel Usage and Emissions 13
Table S16: Leg 3, Scenario 1 Fuel Usage and Emissions 14
Table S17: Leg 3, Scenario 2 Fuel Usage and Emissions 15
Table S18: Leg 3, Scenario 3 Fuel Usage and Emissions 15
Table S19: Leg 3, Scenario 3 Eurotunnel Fuel Usage and Emissions 16
Table S20: Ripening Chamber Fuel Usage and Emissions 17
Table S21: Carbon Sequestration and Indicator Factors for Avocados 18
Table S22: Impact of large-scale use of renewable energy 23
Table S23: Estimated solar power required for 100% renewable energy for farm and packhouse 24
Table 24: Impact of large-scale use of renewable energy 24

Table S25: Uncertainty Assessment Methods and Parameters for Various Life Cycle Phases in Agricultural Production 27
Table S26: Carbon Sequestration Values (Shrublands and Savannahs) 28
Table S27: Carbon Sequestration Values (Composting) 28
Table S28: Monte Carlo Simulation Results for Shipping Variation Uncertainty 29
Table S29: Ship uncertainty scenarios 32
Table S30: Monte Carlo Simulation Results for Shipping Variation Uncertainty 32
Table S31: Uncertainty analysis Results - All Life Cycle Phases 34
Table S32: Comparison of avocado carbon footprint for different life cycle phases and on a per-calorie basis 37
Table S33: Data Quality Matrix (adapted from Blaauw et al., 2020) 38
Table S34: Data Quality Indicator scores for indicators used in this study 38

S1. Key Input data and Assumptions

Table S1: Emissions per Mega Joule Energy

Indicator	Unit	Electricity – South Africa	Electricity - Netherland	Electricity - UK	Electricity - France	Renewable Energy - Solar
Carbon Dioxide Emissions	kg CO_2e/MJ	0.288	0.108	0.059	0.016	0.002
**Indicator**	**Unit**	**Renewable weighted average (solar and wind 50:50)**	**Petrol**	**Diesel**	**Marine Fuel**	**Aviation Fuel**
Carbon Dioxide Emissions	kg CO_2e/MJ	1.67E-03	0.072	0.075	0.080	0.072

Table S2: Fuel Usage Rates

Indicator	Unit	Tractor	Medium Distance Haul	Long Distance Haul
Fuel Usage Rates	Litre/km	0.166		
	Litre/tonne-km		0.018	0.014

Table S3: Assumptions related to Avocado Transportation and Farming

Category	Value	Unit
Avocado weight (including skin and seed)	0.15	kg
Avocado weight (flesh only)	0.1	kg
Avocados/twenty-foot equivalent unit (TEU)	160000	avocados/TEU
Weight/TEU	24000	kg/TEU
Number of avocados/tonne of avocados	6666.67	avocados/tonne
Total farm hectares	7712	hectares
Total farm avocado hectares	760	hectares
Proportion attributed to avocados	19.71%	avocado hectares/farm
Avocado production per hectare per year including waste	9.5	tonnes/ha/year (including waste)
Avocado production per hectare per year excluding waste	8.93	tonnes/ha/year (excluding waste)

Table S4: Fuel Indicator Factors

Diesel Indicator Factors		
Calorific value	MJ/kg Diesel	42.600
Emissions	kg CO_2e/kg Diesel	3.208
Emissions	kg CO_2e/litre Diesel	2.705
Diesel density	kg/litre	0.835
**Petrol Indicator Factors**		

Calorific value	MJ/kg Petrol	43.800
Emissions	kg CO_2e/kg Petrol	3.154
Emissions	kg CO_2e/litre Petrol	2.340
Petrol density	kg/litre	0.742
**Marine Fuel Indicator Factors**		
Calorific value	MJ/kg Marine Fuel	40.800
Emissions	kg CO_2e/kg Marine Fuel	3.250
Marine Fuel Density	kg/litre	0.900
**Jet Fuel Indicator Factors**		
Calorific value	MJ/kg Jet Fuel	43.900
Emissions	kg CO_2e/kg Jet Fuel	3.181
Marine Fuel Density	kg/litre	0.800

Table S5: Fertiliser Indicator Factors

**Nitrate**		
Emissions	kg CO_2e/kg	0.750
Energy oil equivalent	kg Oil Eq/kg	0.620
Energy	MJ/kg	26.760
Water use	Water (litres)/kg	7.510
**Compound (Phorphorous & Potassium)**		
Emissions	kg CO_2e/kg	0.860
Energy oil equivalent	kg Oil Eq/kg	0.410
Energy	MJ/kg	17.680
Water use	Water (litres)/kg	18.600
**Lime**		
Emissions	kg CO_2e/kg	0.048
Energy	MJ/kg	0.243
Water use	Water (litres)/kg	0.055

Table S6: Pesticide Indicator Factors

**Indicator**	**Fungicide**		**Insecticide**		**Growth Regulator**	
Energy use	MJ/kg fungicide	423.00	MJ/kg insecticide	274.00	MJ/kg growth regulator	276.00
Carbon Emissions	kg CO_2e/kg fungicide	29.19	kg CO_2e/kg insecticide	18.91	kg CO_2e/kg growth regulator	19.04
Water use	l/kg fungicide	1.00	l/kg insecticide	1.00	l/kg growth regulator	1.00
	**Herbicide**		**Molluscide**		**Seed treatment**	
Energy use	MJ/kg herbicide	386.00	MJ/kg molluscide	154.00	MJ/kg seed treatment	511.00
Carbon Emissions	kg CO_2e/kg herbicide	26.63	kg CO_2e/kg molluscide	10.63	kg CO_2e/kg seed treatment	35.26
Water use	l/kg herbicide	1.00	l/kg molluscide	1.00	l/kg seed treatment	1.00

Table S7: Farming Enterprise Commodities

Commodity	Hectare
Tomatoes	51.40%
Avocado	19.71%
Mango	3.27%
Onion	6.30%
Forestry	19.32%
**Total**	**100%**
Proportion attributed to avocados	
Total excluding Forestry (not relevant to Packhouse)	80.68%

Table S8: Yield and Energy Use on Farm (various locations excluding packhouses)

Region/Plantation	Hectare		Tonne product marketed per PU	Tonne waste per PU	kWh component	kWh/tonne	Commodity
	Planted	In Production per production unit (PU)					
Greater Tzaneen 1	158.53	141.32	1,371.00	168.50	463,974	368	Avocado
Greater Tzaneen 2	145.98	131.66	384.61	22.59	268,707	229	Avocado
Greater Tzaneen 3	80.49	71.85	474.14	29.06	81,826	128	Avocado
Greater Tzaneen 4	162.82	153.61	2,121.15	113.85	126,102	92	Avocado
Greater Tzaneen 5	11.48	10.87	200.84	9.66	83,999	865	Avocado
Greater Tzaneen 6	66.69	60.59	176.38	32.92	30,554	56	Avocado
Greater Tzaneen 7	16.97	13.58	130.08	8.02	49,080	405	Avocado
Greater Tzaneen 8	9.53	9.08	23.60	1.10	10,069	124	Avocado
Greater Tzaneen 9	12.74	11.99	35.78	1.92	54,817	512	Avocado

S2. Farm Life Cycle Inventory

This section details the LCI for the farm life cycle phase with all relevant data provided.

Table S9: Farm Fuel Usage

**Diesel Use**		
**Indicator**	**Value**	**Unit**
Diesel use	1450.970	litres/hectare/year
Avocados per hectare per year	8930.000	kg avocado/hectare/year
Diesel per kg avocado	0.162	litre/kg avocado/hectare/year
Diesel per tonne avocado	81.370	litre/tonne avocado/hectare/year
	67.944	kg Diesel/tonne avocado/hectare/year
**Per tonne avocados**		
Energy usage	2894.412	MJ/tonne
Emissions	217.964	kg CO_2e/tonne
**Per avocado (150 grams)**		
Energy usage	434.162	kJ/avocado
Emissions	32.695	g CO_2e/avocado
**Petrol Use**		
**Indicator**	**Value**	**Unit**
Petrol use	28.620	litres/hectare/year
Avocados per hectare per year	8930.000	kg avocados/hectare/year
Petrol per kg avocado	0.003	litre/kg avocado/hectare/year
Petrol per tonne avocado	1.600	litre/tonne avocado/hectare/year
	1.187	kg Petrol/tonne avocado/hectare/year
**Per tonne avocados**		
Energy usage	52.068	MJ/tonne
Emissions	3.749	kg CO_2e/tonne
**Per avocado (150 grams)**		
Energy usage	7.810	kJ/avocado
Emissions	0.562	g CO_2e/avocado
**Aviation Fuel Use**		
**Indicator**	**Value**	**Unit**
Aviation fuel use	30.870	tonne/farm
Percentage for avocados	19.7%	
Total tonne assumed used for avocado	8.005	kg aviation fuel/hectare/year
Avocados per hectare per year	8930	kg avocados/hectare/year
Aviation fuel per avocado	0.896	litre aviation fuel/tonne avocados
**Per tonne avocados**		
Energy use	39.356	MJ/tonne
Emissions	2.852	kg CO_2e/tonne
**Per avocado (150 grams)**		
Energy usage	5.903	kJ/avocado
Emissions	0.428	g CO_2e/avocado

Table S10: Pesticide Usage

Fertiliser Usage		
**Indicator**	**Value**	**Unit**
Nitrogen	852.852	tonne N/farm
Compound	1409.102	tonne C/farm
Percentage for avocados	19.7%	
Nitrogen per tonne avocados	3.510	kg N/tonne avocados/year
Compound per tonne avocados	3.300	kg C/tonne avocados/year
**Per tonne avocados**		
Energy use	152.287	MJ/tonne
Emissions	5.481	kg CO_2e/tonne
Water	87.740	l/tonne
**Per avocado**		
Energy usage	22.843	kJ/avocado
Emissions	0.822	g CO_2e/avocado
Water	0.013	l/avocado
**Fungicide Usage**		
**Indicator**	**Value**	**Unit**
Fungicide per tonne avocado	0.88	kg F/tonne avocado (specific data obtained)
**Per tonne avocados**		
Energy use	372.24	MJ/tonne
Emissions	25.68	kg CO_2e/tonne
Water use	0.88	l/tonne
**Per avocado**		
Energy usage	55.836	kJ/avocado
Emissions	3.852684	g CO_2e/avocado
Water	1.32E-04	l/avocado
**Insecticide Usage**		
**Indicator**	**Value**	**Unit**
Insecticide per tonne avocado	0.035	kg I/tonne avocado (specific data obtained)
**Per tonne avocados**		
Energy use	9.590	MJ/tonne
Emissions	0.662	kg CO_2e/tonne
Water use	0.035	l/tonne
**Per avocado**		
Energy usage	1.439	kJ/avocado
Emissions	0.099	g CO_2e/avocado
Water	5.25E-06	l/avocado
**Herbicide Usage**		
**Indicator**	**Value**	**Unit**
Herbicide per tonne avocado	0.022	kg H/tonne avocado (specific data obtained)
**Per tonne avocados**		
Energy use	8.474	MJ/tonne
Emissions	0.585	kg CO_2e/tonne
Water	0.022	l/tonne
**Per avocado**		

Energy usage	1.271	kJ/avocado
Emissions	0.088	g CO_2e/avocado
Water	3.29E-06	l/avocado
**Lime Usage**		
**Indicator**	**Value**	**Unit**
Total tonne used on farm	2354.052	tonne/farm
Percentage for avocados	19.70%	
Lime per tonne avocado	0.007	tonne Lime/tonne avocado
	6.553	kg Lime/tonne avocado
**Per tonne avocados**		
Energy use	1.592	MJ/tonne
Emissions	0.314	kg CO_2e/tonne
Water	0.359	l/tonne
**Per avocado**		
Energy usage	0.239	kJ/avocado
Emissions	0.047	g CO_2e/avocado
Water	5.39E-05	l/avocado

Table S11: Electricity usage for the Farm and Processing Packhouse

**Electricity Usage on Farm**		
**Indicator**	**Value**	**Unit**
Energy obtained from the national grid	100%	
Yearly energy per tonne product	319.000	kWh/tonne product (normalised for all agricultural products)
**Per tonne avocados**		
Energy usage	1148.400	MJ/tonne
Emissions	330.739	kg CO_2e/tonne
**Per avocado (150 grams)**		
Energy usage	172.260	kJ/avocado
Emissions	49.611	kg CO_2e/avocad0
**Packhouse Total Yearly Energy Usage (Including On-Site Refrigeration)**		
**Indicator**	**Value**	**Unit**
Total yearly energy usage	291898.000	kWh/year
Tonne avocado processed	7640.055	tonne/year
Energy obtained from the national grid	100%	
Yearly energy per tonne product	38.206	kWh/tonne product
**Per tonne avocados**		
Energy usage	137.543	MJ/tonne
Emissions	39.612	kg CO_2e/tonne
**Per avocado (150 grams)**		
Energy usage	20.631	kJ/avocado
Emissions	5.942	kg CO_2e/avocado

Table S12: Packaging Energy and Emissions Usage

Indicator	Value	Unit
Box Specification (400x200x145mm)	Most commonly used box on site	
Weight per box	0.316	kg/box
Avocados per box	27	avocados/box
Boxes per tonne avocados	246.914	Boxes/tonne
Weight per tonne avocados	78.025	kg/tonne avocados
**Per tonne avocados**		
Energy usage	1711.003	MJ/tonne
Emissions	64.058	kg CO_2e/tonne
**Per avocado**		
Energy usage	2.57E-01	MJ/avocado
Emissions	9.61E-03	kg CO_2e/avocado

S3. Transportation Life Cycle Inventory

This section details the LCI for the transportation life cycle phase with all relevant data provided. The transportation phase is divided into three legs:

Leg 1 – Long haul trucks from the packhouse in Tzaneen, Limpopo to the Port of Cape Town for export by sea;

Leg 2 – Transportation by sea from the Port of Cape Town to the Port of Rotterdam, and

Leg 3 – Long distance haul from the Port of Rotterdam to the various distribution centres following three scenarios:

Leg 3, scenario 1: Local distribution within Rotterdam,

Leg 3, scenario 2: Local European distribution to Paris, France, and

Leg 3, scenario 3: International distribution to Nottingham, England through utilisation of the Euro Tunnel.

Table S13: Leg 1 Fuel Usage and Emissions

Leg 1 - Long Distance Haul to Cape Town					
**Transportation**			**Refrigeration**		
**Indicator**	**Value**	**Unit**	**Indicator**	**Value**	**Unit**
Haul load	24	tonne avocados	Haul time	20	hours
Total transport distance	1810	km	Energy use	2.7	kW/TEU
Ineffective transport	2%	To compensate for loading and unloading activities	Refrigeration power generated from auxilary power supply	100%	
			Efficiency of system	90%	
**Per tonne avocados**					
Fuel usage	0.014	l/tonne-km	Energy use per journey	54.00	kWh/TEU journey
	25.340	l/tonne		194.40	MJ/TEU journey
Compensate for ineffectiveness	25.847	l/tonne	Compensate for 90% efficiency	213.84	MJ/TEU journey
Energy usage	919.397	MJ/tonne	Energy usage	8.91	MJ/tonne
Emissions	69.235	kg CO_2e/tonne	Emissions	0.67	kg CO_2e/tonne
**Per avocado**					
Energy usage	1.38E-01	MJ/avocado	Energy usage	1.34E-03	MJ/avocado
Emissions	1.04E-02	kg CO_2e/avocado	Emissions	1.01E-04	kg CO_2e/avocado

Table S14: Leg 2 Fuel Usage and Emissions

Leg 2 - Cape Town to Rotterdam by Sea		
**Indicator**	**Value**	**Unit**
Shipping Distance	12500.000	km
Speed (18 knots)	31.328	km/hr
Fuel usage (110 tons/day)	6.667	ton/hr
Travel time	399.000	hours
	16.625	days
TEUs on Akadimos	9126.000	
Fuel usage per ship	2660.000	tonne/journey
Fuel usage per TEU	291.475	kg fuel/TEU journey
Miscellaneous activities (load/unload)	1%	
Ineffectiveness	50%	
**Per tonne avocados**		
Fuel usage	12.145	kg fuel/tonne avo
Compensate for miscellaneous	12.266	kg fuel/tonne avo
Compensate for ineffectiveness	18.399	kg fuel/tonne avo
Energy usage	750.694	MJ/tonne avo
Emissions	59.798	kg CO_2e/tonne avo
**Per avocado**		
Energy usage	1.13E-01	MJ/avocado
Emissions	8.97E-03	kg CO_2e/avocado

Table S15: Leg 2 Refrigeration Fuel Usage and Emissions

Leg 2 - Refrigerate at Cape Town Port		
**Indicator**	**Value**	**Unit**
Refrigeration Time	72	hours
Energy use	2.7	kW/TEU
Energy obtained from National Grid	100%	South African grid
Efficiency of system	50%	
**Per tonne avocados**		
Energy use per journey	194.400	kWh/TEU
	699.840	MJ/TEU
Compensate for efficiency	1049.760	MJ/TEU
Energy usage	43.740	MJ/tonne
Emissions	12.597	kg CO_2e/tonne
**Per avocado**		
Energy usage	6.56E-03	MJ/avocado
Emissions	1.89E-03	kg CO_2e/avocado
**Leg 2 - Refrigerate on Ship**		
**Indicator**	**Value**	**Unit**
Refrigeration Time	399	hours
Energy use	2.7	kW/TEU
Energy obtained from National Grid	100%	Onboard vessel
Efficiency of system	50%	
**Per tonne avocados**		

Energy use per journey	1077.300	kWh/TEU
	3878.280	MJ/TEU
Compensate for efficiency	5817.420	MJ/TEU
Energy usage	242.393	MJ/tonne
Emissions	19.308	kg CO_2e/tonne
**Per avocado**		
Energy usage	3.64E-02	MJ/avocado
Emissions	2.90E-03	kg CO_2e/avocado
**Leg 2 - Refrigerate at Rotterdam**		
**Indicator**	**Value**	**Unit**
Refrigeration Time	48	hours
Energy use	2.7	kW/TEU
Energy obtained from National Grid	100%	Dutch grid
Efficiency of system	50%	
**Per tonne avocados**		
Energy use per journey	129.600	kWh/TEU
	466.560	MJ/TEU
Compensate for efficiency	699.840	MJ/TEU
Energy usage	29.160	MJ/tonne
Emissions	3.149	kg CO_2e/tonne
**Per avocado**		
Energy usage	4.37E-03	MJ/avocado
Emissions	4.72E-04	kg CO_2e/avocado

Table S16: Leg 3, Scenario 1 Fuel Usage and Emissions

**Leg 3 - Scenario 1 - From Distribution Centre to Rotterdam**					
**Transportation**			**Refrigeration**		
**Indicator**	**Value**	**Unit**	**Indicator**	**Value**	**Unit**
Haul load	24	tonne avocados	Haul time	1	hours
Total transport distance	50	km	Energy use	2.7	kW/TEU
Ineffective transport	0%		Efficiency of system	90%	
**Per tonne avocados**					
Fuel usage	0.014	l/tonne-km	Energy use per journey	2.700	kWh/TEU journey
	0.700	l/tonne		9.720	MJ/TEU journey
Compensate for ineffectiveness	0.700	l/tonne	Compensate for 90% efficiency	10.692	MJ/TEU journey
Energy usage	24.900	MJ/tonne	Energy usage	0.446	MJ/tonne
Emissions	1.875	kg CO_2e/tonne	Emissions	0.034	kg CO_2e/tonne
**Per avocado**					
Energy usage	3.73E-03	MJ/avocado	Energy usage	6.68E-05	MJ/avocado

Emissions	2.81E-04	kg CO_2e/avocado	Emissions	5.03E-06	kg CO_2e/avocado

Table S17: Leg 3, Scenario 2 Fuel Usage and Emissions

Leg 3 - Scenario 2 - From Distribution Centre to France					
**Transportation**			**Refrigeration**		
**Indicator**	**Value**	**Unit**	**Indicator**	**Value**	**Unit**
Haul load	24	tonne avocados	Haul time	6	hours
Total transport distance	450	km	Energy use	2.7	kW/TEU
Ineffective transport	0%		Efficiency of system	90%	
**Per tonne avocados**					
Fuel usage	0.014	l/tonne-km	Energy use per journey	16.200	kWh/TEU journey
	6.300	l/tonne		58.320	MJ/TEU journey
Compensate for ineffectiveness	6.300	l/tonne	Compensate for 90% efficiency	64.152	MJ/TEU journey
Energy usage	224.097	MJ/tonne	Energy usage	2.673	MJ/tonne
Emissions	16.876	kg CO_2e/tonne	Emissions	0.201	kg CO_2e/tonne
**Per avocado**					
Energy usage	3.36E-02	MJ/avocado	Energy usage	4.01E-04	MJ/avocado
Emissions	2.53E-03	kg CO_2e/avocado	Emissions	3.02E-05	kg CO_2e/avocado

Table S18: Leg 3, Scenario 3 Fuel Usage and Emissions

Leg 3 - Scenario 3 - From Distribution Centre to England (Including Eurotunnel)					
**Transportation**			**Refrigeration**		
**Indicator**	**Value**	**Unit**	**Indicator**	**Value**	**Unit**
Haul load	24	tonne avocados	Haul time	12	hours
Total transport distance	700	km	Energy use	2.7	kW/TEU
Ineffective transport	0%		Efficiency of system	90%	
**Per tonne avocados**					
Fuel usage	0.014	l/tonne-km	Energy use per journey	32.400	kWh/TEU journey
	9.800	l/tonne		116.640	MJ/TEU journey
Compensate for ineffectiveness	9.800	l/tonne	Compensate for 90% efficiency	128.304	MJ/TEU journey
Energy usage	348.596	MJ/tonne	Energy usage	5.346	MJ/tonne
Emissions	26.251	kg CO_2e/tonne	Emissions	0.403	kg CO_2e/tonne

Per avocado					
Energy usage	5.23E-02	MJ/avocado	Energy usage	8.02E-04	MJ/avocado
Emissions	3.94E-03	kg CO_2e/avocado	Emissions	6.04E-05	kg CO_2e/avocado

Table S19: Leg 3, Scenario 3 Eurotunnel Fuel Usage and Emissions

Leg 3 - Scenario 3 - Eurotunnel		
**Indicator**	**Value**	**Unit**
Haul load	24	tonne avocados
Total transport distance	50	km
Ineffective transport	0%	
Average freight emissions per crossing	8.8	kgCO_2e/TEU
**Per tonne avocados**		
Energy usage	6.217	MJ/tonne
Emissions	0.367	kg CO_2e/tonne
**Per avocado**		
Energy usage	9.33E-04	MJ/avocado
Emissions	5.50E-05	kg CO_2e/avocado

S4. Ripening Chamber Life Cycle Inventory

This section details the LCI for the ripening chamber life cycle phase with all relevant data provided.

Table S20: Ripening Chamber Fuel Usage and Emissions

Netherlands and France		
**Indicator**	**Value**	**Unit**
Ripening period	28.320	hours
Avocados per chamber	160000.000	avocados/chamber
	24.000	tonne/chamber
Energy per hour for cooling chamber	3.600	MJ/hr
Total energy for cooling	101.952	MJ/total period
Energy per hour for ethylene generator	21.100	MJ/hr
Energy for ethylene production	26.000	GJ/tonne
Energy for 3 litres ethylene	78.000	MJ/3 kg
Total energy usage	201.052	MJ/24 tonne
	8.377	MJ/tonne

Netherlands			France		
**Per tonne avocados**					
Energy usage	201.052	MJ/tonne	Energy usage	201.152	MJ/tonne
Emissions	21.714	kg CO_2e/tonne	Emissions	3.129	kg CO_2e/tonne
**Per avocado**					
Energy usage	3.02E-02	MJ/avocado	Energy usage	3.02E-02	MJ/avocado
Emissions	3.26E-03	kg CO_2e/avocado	Emissions	4.69E-04	kg CO_2e/avocado

S5. Carbon Sequestration Life Cycle Inventory

Table S21: Carbon Sequestration and Indicator Factors for Avocados

**Compost - Dairy Manure and Green Waste**		
Carbon sequestration potential (A)	0.690	kg CO_2e/kg Compost (Vergara and Silver, 2019)
Total tonne used on farm	34179.409	mega tonne/farm
Tonne waste from fruit	11784.755	mega tonne/farm
Total for farm minus waste (X)	22394.654	tonne
Total farm area inclusive of forestry (Y)	3856	hectare
Total tonne per hectare Avocado	5.808 (= X / Y)	tonne compost/hectare avocado
Compost per Avocado tonne (8.93 tonne average yield per hectare excluding waste of 6%) (Z)	0.650	Tonne compost/tonne avocado
**Carbon Offset per Tonne Avocado**	**0.449 (=A × Z)**	**tonne CO_2e Sequestrated/tonne avocado**
**Shrubland**		
Carbon sequestration potential (A)	1.900	tonne CO_2e/hectare shrubland/year (Lou et al., 2007)
Total Shrubland area for Farm	220.000	hectare
Attributed towards Avocado (19.71% - See Table S3) (X)	43.361	hectares for avocados
Total hectares of avocados (Y)	760	hectares
Hectare Shrubland per hectare Avocado (Z)	0.057 (=X/Y)	hectare shrubland/hectare avocado
Carbon sequestration per hectare avocado (8.93 tonne average yield per hectare excluding waste of 6%)	0.108 (=A × Z)	tonne CO_2e/hectare avocado
**Carbon Offset per Tonne Avocado**	**0.012**	**tonne CO_2e/tonne avocado**
**Preserved Savannah**		
Carbon sequestration potential (A)	0.350	tonne CO_2e/hectare savannah/year (Zhou et al., 2022)
Total Savannah area	6000.000	hectare
Percentage towards Avocado (19.71% - See Table S3) (X)	1182.573	hectare for avocados
Total hectares of avocados (Y)	760	hectares
Hectare Savannah per hectare Avocado (Z)	1.556 (= X / Y)	Hectare savannah per hectare avocado
Carbon sequestration per hectare avocado (8.93 tonne average yield per hectare excluding waste of 6%)	0.545 (= A × Z)	tonne CO_2e/hectare avocado
**Carbon Offset per Tonne Avocado**	**0.061**	**tonne CO_2e/tonne avocado**

For a list of secondary input data please see Table S26 and Table S27.

S5.1 Description of Calculation Model Represented in Table S21

1. Compost - Dairy Manure and Green Waste

Carbon Sequestration Potential (A):

- Value: 0.690 kg CO2e per kg compost
- Source: Vergara and Silver (2019)

Total Compost Used on Farm:

- Value: 34,179.409 megatonnes per farm

Tonne Waste from Fruit:

- Value: 11,784.755 megatonnes per farm

Total Compost for Farm Minus Waste (X):

- Calculation:

$$Total\ Compost\ Used - Tonne\ Waste\ from\ Fruit$$

- Value:

$$34{,}179.409 - 11.784.755 = 22{,}394.654\ tonnes$$

Total Farm Area Inclusive of Forestry (Y):

- Value: 3,856 hectares

Total Tonne per Hectare Avocado:

- Calculation:

$$\frac{Total\ Compost\ for\ Farm\ Minus\ Waste\ (X)}{Total\ Farm\ Area\ Inclusive\ of\ Forestry\ (Y)}$$

- Value:

$$\frac{22{,}394.654}{3{,}856} \approx 5.808\ tonnes\ compost/hectare\ avocado$$

Compost per Tonne Avocado (Z):

- Calculation:

$$\frac{Total\ Tonne\ per\ Hectare\ Avocado}{Average\ Yield\ per\ Hectare\ excluding\ 6\%\ waste}$$

- Average Yield per Hectare excluding waste: 8.93 tonnes
- Value:

$$\frac{5.808}{8.93} \approx 0.650\ tonnes\ compost/tonne\ avocado$$

Carbon Offset per Tonne Avocado:

- Calculation:

  $Carbon\ Sequestration\ Potential\ (A) \times Compost\ per\ Tonne\ Avocado\ (Z)$

- Value:

$$0.690\ \times 0.650\ \approx 0.499\ tonnes\ CO2e\ sequestered/tonne\ avoado$$

2. Shrubland

Carbon Sequestration Potential (A):

- Value: 1.900 tonnes CO2e per hectare shrubland per year
- Source: Lou et al. (2007)

Total Shrubland Area for Farm:

- Value: 220 hectares

Hectares Attributed towards Avocado (X):

- Calculation:

$$Total\ Shrubland\ Area\ for\ Farm\ \times Fraction\ Land\ Attributed\ to\ Avocado\ (Table\ S3)$$

- Value:

$$220\ \times 0.1971\ \approx 43.361\ hectares$$

Total Hectares of Avocados (Y):

- Value: 760 hectares

Hectare Shrubland per Hectare Avocado (Z):

- Calculation:

$$\frac{Attributed\ towards\ Avocado\ (X)}{Total\ Hectares\ of\ Avocados\ (Y)}$$

- Value:

$$\frac{43.361}{760}\ \approx 0.057\ hectare\ shrubland/hectare\ avocado$$

Carbon Sequestration per Hectare Avocado:

- Calculation:

$$Carbon\ Sequestration\ Potential\ (A) \times Hectare\ Shrubland\ per\ Hectare\ Avocado\ (Z)$$

- Value:

$$1.900\ \times 0.057\ \approx 0.108\ tonnes\ CO_2e/heactare\ avocado$$

Carbon Offset per Tonne Avocado:

Calculation:

$$\frac{Carbon\ Sequestration\ per\ Hectare\ Avocado}{Average\ Yield\ per\ Hectare\ excluding\ Waste}$$

Value:

$$\frac{0.108}{8.93} \approx 0.012\ tonnes\ CO_2e/tonne\ avocado$$

3. Preserved Savannah

Carbon Sequestration Potential (A):

- Value: 0.350 tonnes CO_2e per hectare savannah per year
- Source: Zhou et al. (2022)

Total Savannah Area:

- Value: 6,000 hectares

Hectares Attributed towards Avocado (X):

- Calculation:

$$Total\ Savannah\ Area\ \times\ Fraction\ Land\ Attributed\ to\ Avocado\ (Table\ S3)$$

- Value:

$$6{,}000\ \times 0.1971\ \approx 1{,}182.573\ hectares$$

Total Hectares of Avocados (Y):

- Value: 760 hectares

Hectare Savannah per Hectare Avocado (Z):

- Calculation:

$$\frac{Hectares\ Attributed\ towards\ Avocado\ (X)}{Total\ Hectares\ of\ Avocados\ (Y)}$$

- Value:

$$\frac{1{,}182.573}{760} \approx 1.556\ hectare\ savannah/hectare\ avocado$$

Carbon Sequestration per Hectare Avocado:

- Calculation:

$$Carbon\ Sequestration\ Potential\ (A) \times Hectare\ Savannah\ per\ Hectare\ Avocado\ (Z)$$

- Value:

$$0.350 \times 1.556 \approx 0.545\ tonnes\ CO_2e/hectare\ avocado$$

Carbon Offset per Tonne Avocado:

- Calculation:

$$\frac{Carbon\ Sequestration\ per\ Hectare\ Avocado}{Average\ Yield\ per\ Hectare\ excluding\ Waste}$$

- Value:

$$\frac{0.545}{8.93} \approx 0.061\ tonnes\ CO_2e/tonne\ avocado$$

Total Carbon Offset per Tonne of Avocados

Combining the contributions from composting, shrubland, and savannah:

Total Carbon Credits per Tonne of Avocados:

- Calculation:

$$0.499\ (Compost) + 0.012\ (Shrubland) + 0.061\ (Savannah)$$

- Value:

$$0.499 + 0.012 + 0.061 = 0.522\ tonnes\ CO_2e\ sequestered\ per\ tonne\ avocado$$

This comprehensive explanation should help clarify the calculations and make the supplementary materials more understandable.

S6. Sensitivity Analysis

Use of renewable energy for the farm and packhouse

Renewable energy, such as solar and wind, presents a viable and environmentally friendly alternative to conventional national grid power. While requiring initial capital investment, the high Internal Rate of Return (IRR) brings forth economic benefits and significantly reduces environmental emissions. In South Africa, solar and wind are the primary forms of renewable energy used by the private sector (IPCC, 2022). Although the electricity generation of renewables itself is emission-free, upstream processes contribute to their overall carbon footprint. This study assumes 1.67E-03 kg CO_2e/MJ for solar electricity and 1.11E-03 kg CO_2e/MJ for wind (Pehl et al., 2017). The sensitivity analysis evaluates the scenario where the farming enterprise's packhouse operations exclusively use solar electricity, considering a 100% renewable energy scenario compared to the 'business-as-usual' approach. Wind-generated renewable energy is not considered based on discussions with the relevant farming enterprise. The results of using 100% renewable energy compared to the 'business-as-usual' scenario are outlined in Table S22.

Table S22: Impact of large-scale use of renewable energy

**Indicator**	**Unit**	**Business-as-usual**	**Renewable Energy**
**Per tonne product**			
Total Energy Use (Farm & Packhouse)	**kWh/tonne**	357.21	
Total Emissions	**kg CO_2e/tonne**	370.35	2.14

The results show that transitioning to large-scale renewable energy for the packhouse and associated processes can eliminate up to 368.21 kg CO_2e/tonne from the avocado's life cycle carbon footprint. An optimisation assessment calculates the solar panel quantity and land needed for 100% renewable energy operation of the farm and packhouse. Requiring 196.32 watts/tonne daily (1,753.17 watts/hectare), based on an average 5.3 peak sunlight hours in Tzaneen and a 23% efficiency assumption, solar panels considered range from 250W to 400W. These findings suggest that 1.1 to 1.4 hectares of solar panels can power the 1,025 hectares of active avocado plantations, as detailed in Table S23.

Table S23: Estimated solar power required for 100% renewable energy for farm and packhouse

	**Panels required**		**Area required**	
**Solar Panel**	**400W**	**250W**	**400W**	**250W**
**Per hectare avocados**	6 panels	9 panels	11 m^2	14 m^2
**For avocado farm as whole (1025 hectares)**	5 526 panels	8 841 panels	11 052 m^2	14 146 m^2

Delivering avocados unripened (green) vs. artificially ripened

To extend shelf-life, avocados are transported unripened and artificially ripened before reaching retail. Prior findings indicate that artificial ripening contributes up to 5.67% of an avocado's total emissions. Selling avocados unripened, allowing consumers to ripen them naturally, removes the artificial ripening step, achieving a 5.67% emission reduction, equivalent to eliminating up to 21.71 kg CO_2e/tonne of avocados.

Alternatives to cardboard packaging

Cardboard packaging significantly impacts the life cycle of avocados, constituting 7.24% of the mass, with 78 kg shipped per tonne. Despite recycling in Europe, the production-related environmental impacts persist. Alternatives like reusable plastic containers, potentially enhanced with ethylene removal properties, offer sustainability. A sensitivity analysis, shown in Table S15, compares these containers to cardboard boxes. The results, considering extraction, manufacturing, transport, cleaning, and end-of-life (López-Gálvez et al., 2021), depict similar physical characteristics to current cardboard boxes, assuming cleaning occurs after each use at a rate of 150 rotations (uses) before the end of life.

Table 24: Impact of large-scale use of renewable energy

**Indicator**	**Unit**	**Single-use cardboard box**	**Reusable plastic crates**
**Per tonne product**			
Energy use per box	**MJ/box**	6.93	0.71
Emissions per box	**kg CO_2e/box**	0.26	0.02
Energy use per tonne avocado	**MJ/tonne**	1711.00	174.81

Energy use per tonne avocado	**kg CO_2e/tonne**	64.06	5.95

These results indicate a 58.11 kg CO_2e/tonne (90.72%) decrease in emissions from shifting from single use cardboard to reusable plastic containers. However, given the large benefit in reduction of environmental impacts, research has shown reusable plastic crates may increase the risk of cross-contamination when fresh produce is shipped (López-Gálvez et al., 2021) and should be considered.

Reduce wastage

Total waste for the farm is calculated as 590 kg (6.73%) avocados per hectare per year and 30 kg (3%) per tonne transported to Europe. This equates to a combined 848.45 kg (9.85%) avocados per hectare per year lost to spoil, a significant percentage which affects not only economic and environmental metrics, but substantially impacts global food security and is a common percentage of food waste in agriculture globally (WWF, 2021).

Total Optimisation Emission Savings actively pursued by the relevant stakeholders

Utilising the potential optimisation determined for certain life cycle phases, the total life cycle impacts incorporating carbon footprint mitigation measures may be calculated, summarised in life cycle phases previously described, of a typical avocado grown in South Africa and enjoyed in Europe. The total life cycle impact reducing from 382.97 kg CO_2e/tonne to a theoretical -68.54 kg CO_2e/tonne (117.90% reduction), allowing ambitions of Net Zero to be achieved.

S7. Uncertainty Analysis

S7.1 Methodology for Uncertainty Assessment

1. Scenario Uncertainty Analysis

In this step, we explore hypothetical scenarios that encompass variations in critical factors affecting different life cycle phases. By considering a range of plausible conditions, we derive upper and lower quartile values. These values provide insight into potential outcomes under varying circumstances.

2. Monte Carlo Simulations

For phases with substantial datasets, we employ Monte Carlo simulations. These simulations generate numerous iterations, each representing a possible outcome based on the probability distributions of input parameters. Extracting upper and lower quartile values from the Monte Carlo results allows us to comprehensively understand uncertainty within the assessed phases.

3. Data Quality Assessment

To address data quality concerns, we assess the reliability of our dataset. By acquiring updated indicator factor data relevant to the products and processes under scrutiny, we refine our information. Integrating this improved dataset into the Monte Carlo simulations accounts for uncertainties stemming from data quality. The resulting upper and lower quartile values reveal the extent of uncertainty attributable to data quality concerns.

4. Analysis and Comparison

Systematically evaluating the uncertainty ranges represented by upper and lower quartile values across all life cycle phases is crucial. By aggregating these uncertainty ranges, we gauge the overall impact of uncertainty on assessment results. Comparing the uncertainty bounds with the current assessment findings ensures a rigorous evaluation of reliability and robustness.

Table S25: Uncertainty Assessment Methods and Parameters for Various Life Cycle Phases in Agricultural Production

Life Cycle Phase	Uncertainty Method	Uncertainty Parameters	Uncertainty treatment
Liquid fuel use on Farm	Scenario Analysis	Variability in fuel consumption volume	Fluctuate fuel use for inefficiency by ±30%
Fertiliser and Pesticide	Data Quality Assessment	Reliability of indicator factors for fertilizers and pesticides	Apply Monte Carlo simulations to a comprehensive fertilizer and pesticide indicator factor database
Electricity - Farm	Scenario Analysis	Variation in energy consumption per hectare	Fluctuate energy use by ±30% for seasonal changes
Electricity - Packhouse	None	-	-
Packaging	Data Quality Assessment	Accuracy of indicator factors for cardboard boxes	Apply Monte Carlo simulations to a comprehensive cardboard box indicator factor database
Transport by Road	Data Quality Assessment	Precision of truck fuel usage per kilometer	Apply Monte Carlo simulations to a comprehensive database of typical truck fuel usage
Transport by Sea	Scenario Analysis	Fluctuations in marine fuel usage, trip duration, port waiting times, ship size, and ship speed	Utilise 10 scenarios to account for various situations, apply Monte Carlo simulations to scenario database to determine probability density functions
Ripening Chamber	Scenario Analysis	Seasonal variation in energy usage for cooling	Fluctuate energy use for cooling to account for seasonal variation (-100% to +320%)
Carbon Sequestration	Data Quality Assessment	Dependability of indicator factors for carbon sequestration potential	Apply Monte Carlo simulations to a comprehensive carbon sequestration potential per hectare database
Yield	Scenario Analysis	Changes in yield per hectare per year	Utilise 8 scenarios to account for fluctuation in yield per hectare per year (-40% to +40%), apply Monte Carlo simulations

S7.1 Example of Data Quality Assessment Uncertainty – Carbon Sequestration

This section details an example of the data quality assessment for carbon sequestration potential. Table S26 and Table S27 illustrates the database for carbon sequestration potential, followed by the corresponding Probability Density Functions for the database derived from Monte Carlo simulations.

Table S26: Carbon Sequestration Values (Shrublands and Savannahs)

Land use change/measure	Study Location	Net CO_2e Rate	Unit	Reference
Pelargonium - scabrum	UK	90	tonnes C/ ha	Natural England Research Report (2012)
Dwarf shrub (life span of 10 years)	Southern California	1.9	tonnes CO_2e/ha/yr	Luo et al. (2007)
Shrubland - Alpine	China	2.75	tonnes CO_2e/ha/yr	Zhao et al. (2006)
Shrubland	Arizona	7.77	tonnes CO_2e/ha/yr	Scott et al. (2006)
Preserved Bushveld (e.g.Savannah Conservancy)<br>Assumed similar to typical Grassland	Global	7.3	tCO_2e/ha/yr	Boerema, Van der Biest & Miere (2016)
Savannah	South Africa	0.35	tCO_2e/ha/year	Zhou et al. (2022)
Savannah	California	5.17	tCO_2e/ha/yr	Ma et al. (2007)
Alpine Grassland	China	6.34	tCO_2e/ha/yr	Kato et al. (2006)
Savannah	Sub-Saharan Africa	0.1 to 5.3	MgC/ha/year	Vågen et al. (2005)
Grassland	Portugal	6.97	tCO_2e/ha/yr	Aires et al. (2007)
Savannah	East Africa	0.1 to 3.1	MgC/ha/year	Tessema et al. (2020)

Table S27: Carbon Sequestration Values (Composting)

Compoting	Study Location	Net CO_2e Rate	Unit	Reference
Compost - Dairy Manure and Green Waste	California	-690	kg CO_2e/tonne Compost	Vergara and Silver (2019)
Composting	-	-3100	kg CO_2e/tonne Compost	Brown et al. (2008)
Composting	-	-900 to +300	kg CO_2e/tonne Compost	Boldrin et al. (2009)
Composting	California	-41	kg CO_2e/tonne Compost	Nordahl et al. (2020)
Composting	California	-7533.33	kg CO_2e/ha/year	DeLonge et al. (2013)
Composting – Food Waste	USA	-198.42	kg CO_2e/tonne Compost	US EPA (2016)
Composting – Mixed Organics		-176.37	kg CO_2e/tonne Compost	
Composting –Green Waste	USA	-95.48 to -41.29	kg CO_2e/tonne Compost	Levis and Barlaz (2011)
Composting	Italy	+199 to +250	kg CO_2e/tonne Compost	Pergola et al. (2020)

Compost -Green Waste	California	+926	kg CO_2e/tonne Compost	Pérez et al. (2023)
Composting – Food Waste	Italy	+43.1 to 96.3	kg CO_2e/tonne Compost	Castellani et al. (2019)
Composting	Italy	130	kg CO_2e/tonne Compost	Blengini (2008)
Composting	Italy	+162 to +344	kg CO_2e/tonne Compost	Buratti et al. (2015)
Composting	Italy	+11.1 to +209	kg CO_2e/tonne Compost	Colón et al. (2012)
Composting	Turkey	794	kg CO_2e/tonne Compost	Yay (2015)
Composting - Solid Manure	China	1780	kg CO_2e/tonne Compost	Liu et al. (2022)
Composting		+82 to +126	kg CO_2e/tonne Compost	Martínez-Blanco et al. (2010)
Composting	Korea	+51 to +154	kg CO_2e/tonne Compost	Padeyanda et al. (2015)
Composting –Municipal Solid Waste	India	+73.1 to +731.89	kg CO_2e/tonne Compost	Rana et al. (2019)
Composting - OFMSW		+3430 to 4920	kg CO2e/tonne Compost	Yadav and Smadder (2018)
Composting - OFMSW	Sri Lanka	+218	kg CO2e/tonne Compost	Weligama Thuppahige et al. (2022)

Table S28: Monte Carlo Simulation Results for Shipping Variation Uncertainty

MC Results	Lower Quartile (Q1)	This Study	Upper Quartile (Q3)
Shrubland, Savannah Carbon Sequestration (kg CO_2e/hectare/year)	860	400	5750
Composting Carbon Sequestration (kg CO_2e/tonne compost)	-19.93	-690	416.39
Weighted Average Carbon Sequestration (kg CO_2e/tonne avocado)	-167.72	-521.88	-764.09

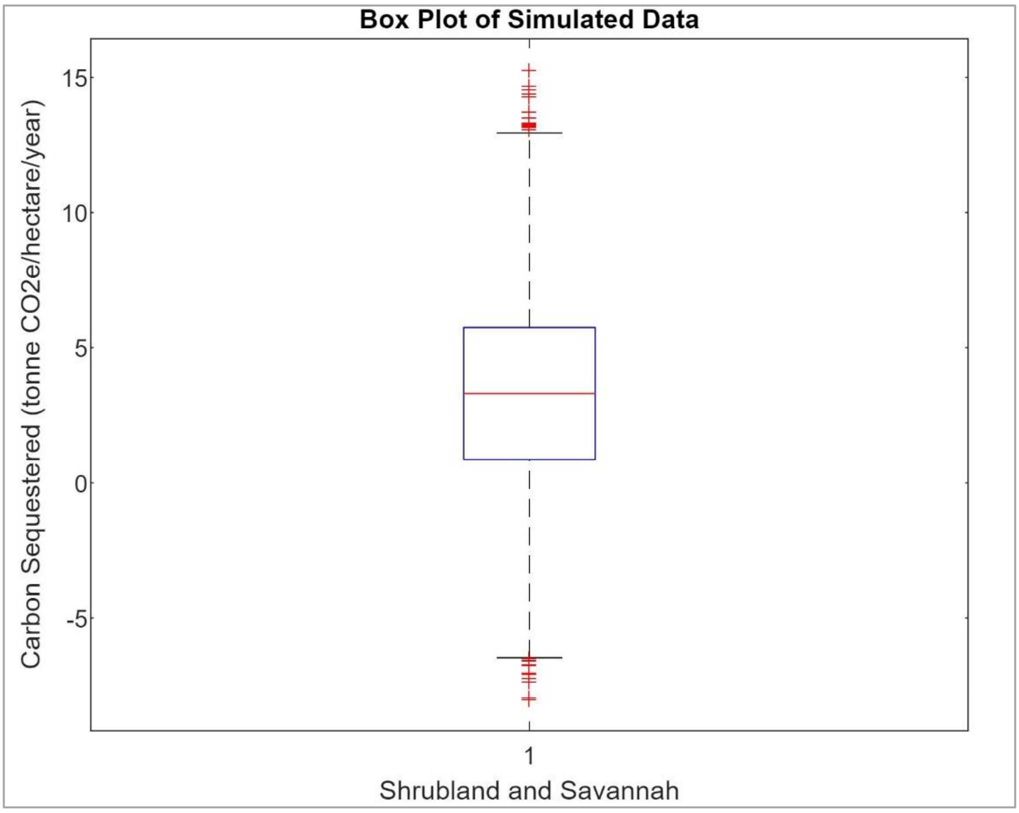

Figure S1: Monte Carlo Results - Box Plot of Uncertainty for Shrubland and Savannah

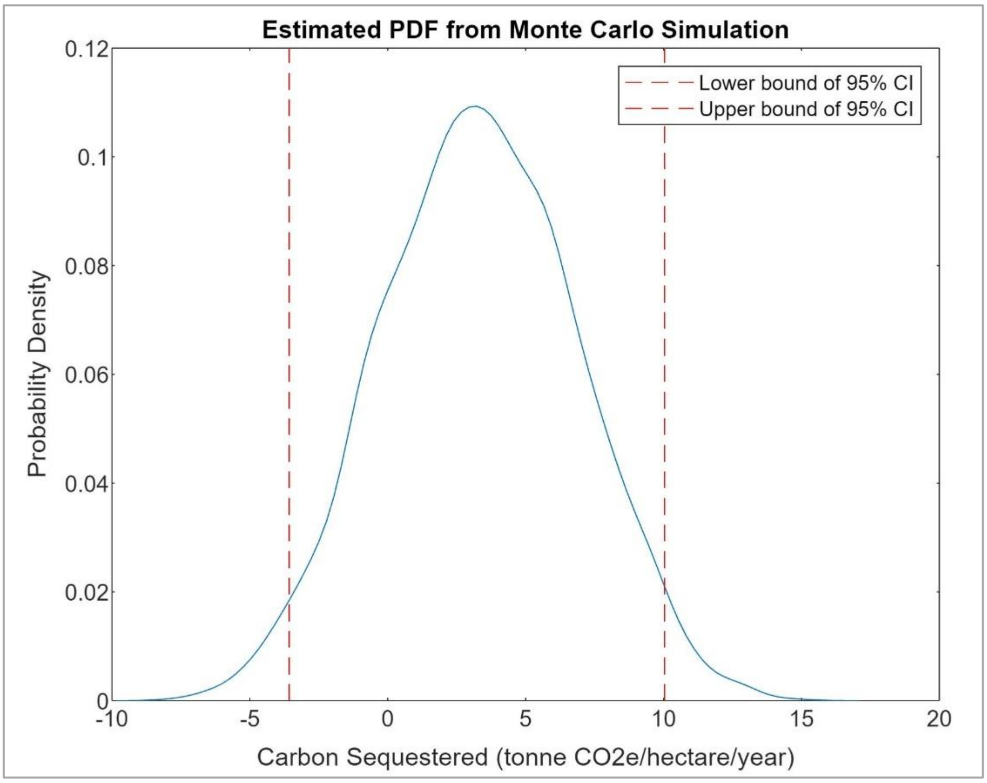

Figure S2: Monte Carlo Results – Probability Density Function of Uncertainty for Shrubland and Savannah

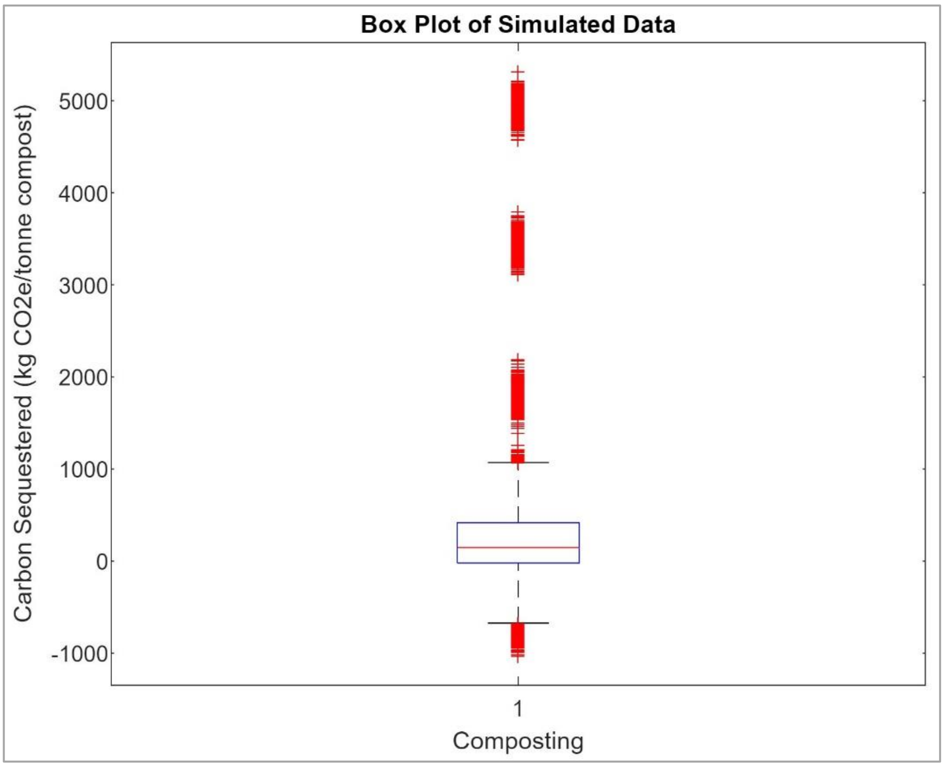

Figure S3: Monte Carlo Results – Box Plot of Uncertainty for Composting

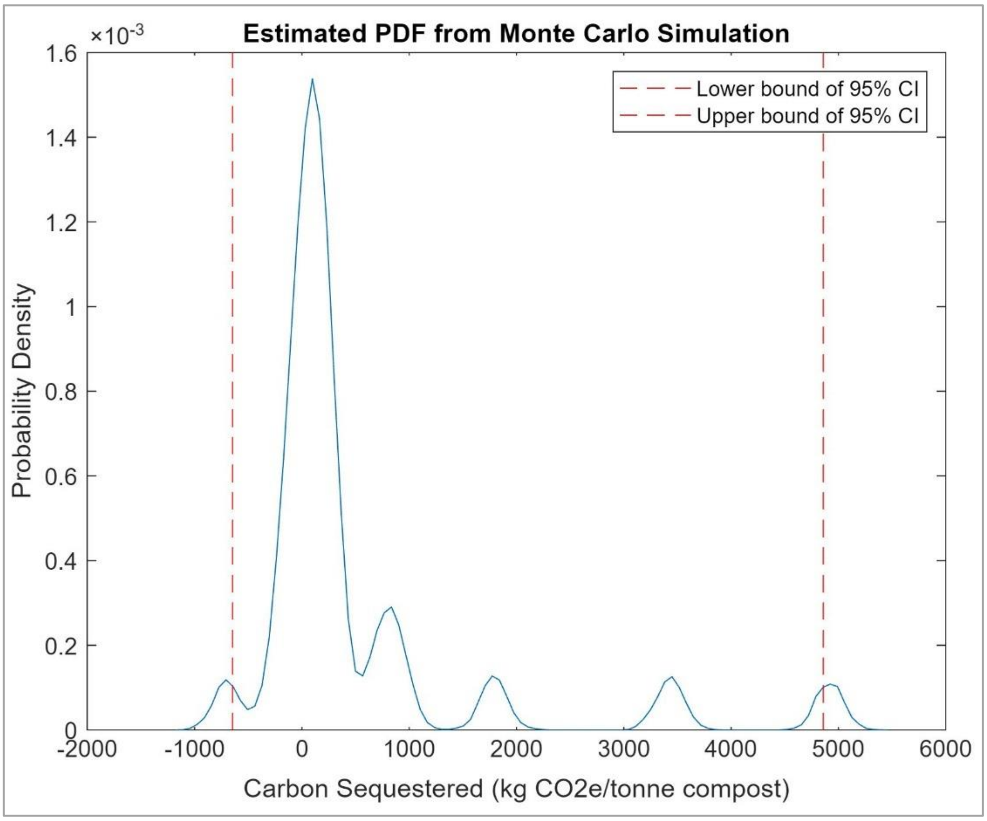

Figure S4: Monte Carlo Results – Probability Density Function of Uncertainty for Compost

S7.2 Example of Scenario Analysis Uncertainty – Farm Energy use per Plantation

Table S29: Ship uncertainty scenarios

Scenario	Travel time (in days)	Days waiting at port (refrigeration)	Ship size (TEU)	Fuel consumption (tonne per day)	kg Fuel/TEU Journey	Refrigeration variations (kg CO_2e/TEU journey)	Shipping variations (kg CO_2e/TEU journey)	Total variation (kg CO_2e/TEU journey)
This study	16.625	3	9126	160	291.47	22.541	59.798	94.936
Scenario 1	13	1	4500	110	317.78	18.312	65.194	87.706
Scenario 2	14	3	5500	120	305.45	19.479	62.666	94.742
Scenario 3	14	4	6500	130	280.00	19.479	57.444	93.719
Scenario 4	20	2	7500	140	373.33	26.477	76.592	111.467
Scenario 5	16	4	8500	150	282.35	21.812	57.926	96.534
Scenario 6	15	3	10500	170	242.86	20.645	49.824	83.066
Scenario 7	11	6	5000	115	253.00	15.980	51.905	93.078
Scenario 8	18	2	8000	145	326.25	24.144	66.932	99.475
Scenario 9	19	4	9500	160	320.00	25.311	65.650	107.757
Scenario 10	20	5	15000	190	253.33	26.477	51.973	99.445
								**MC Uncertainty Input Data**

Table S30: Monte Carlo Simulation Results for Shipping Variation Uncertainty

MC Results	Lower Quartile (Q1)	This Study	Upper Quartile (Q3)
Total Variation (kg CO_2e/TEU journey)	90.63	94.936	101.94

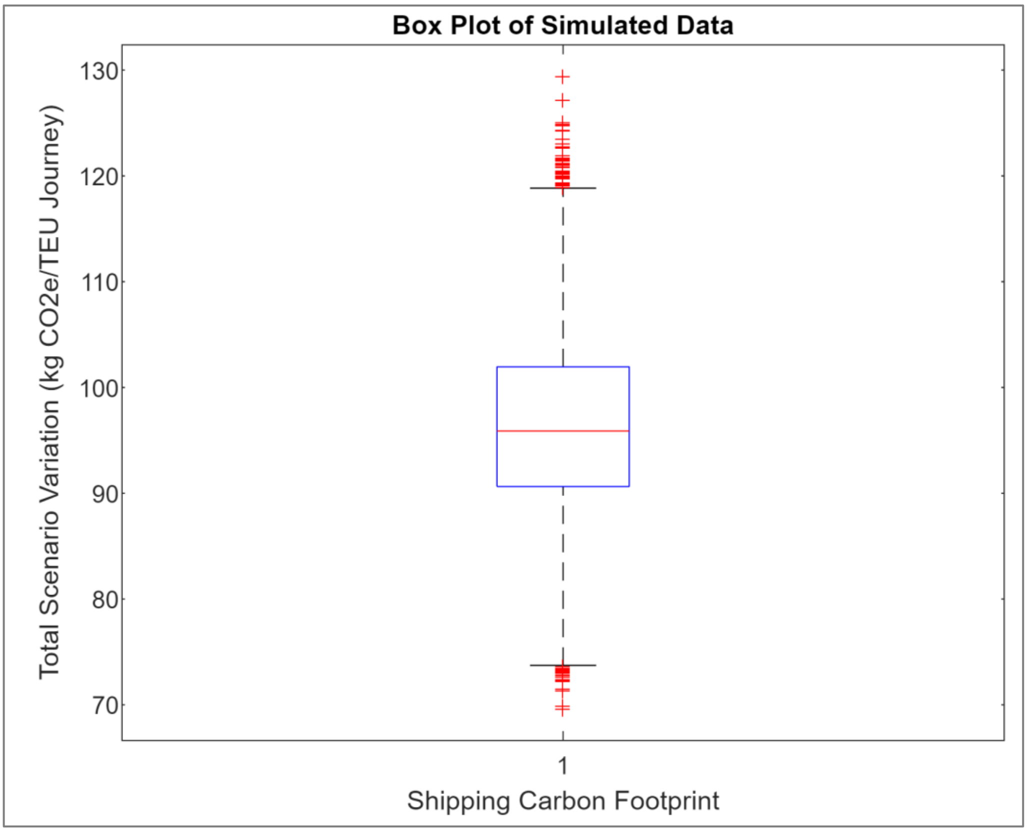

Figure S5: Monte Carlo Results - Box Plot of Uncertainty for Shipping Variation

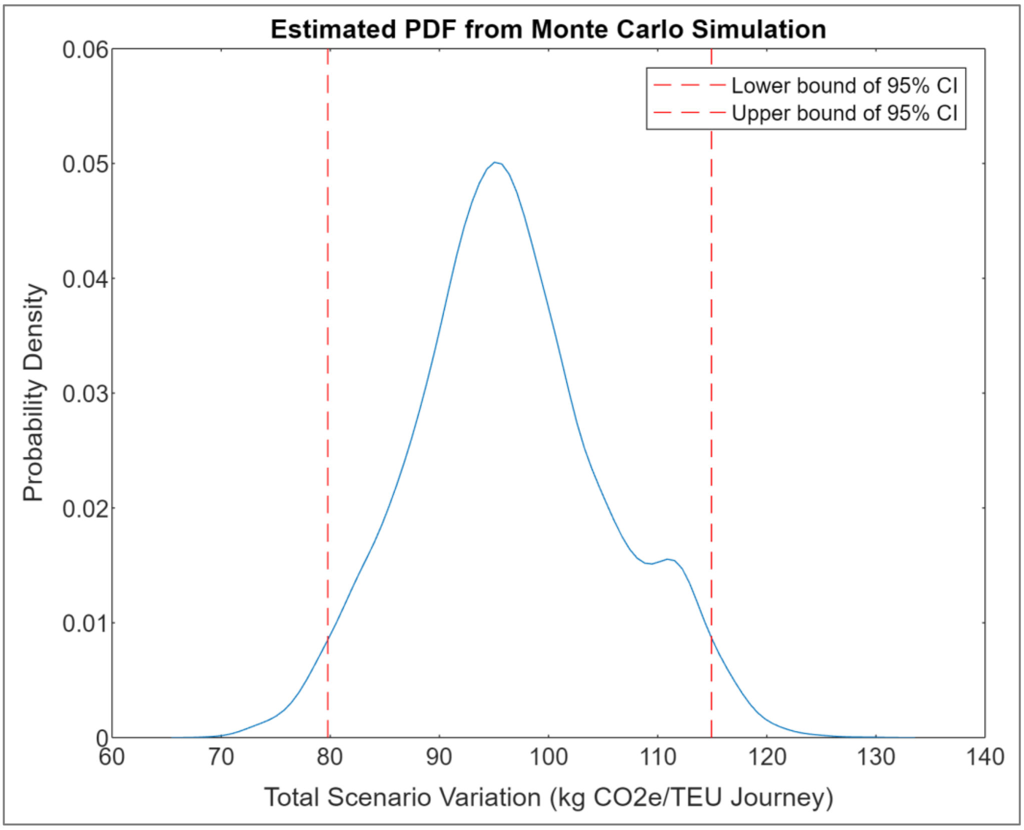

Figure S6: Monte Carlo Results – Probability Distribution Function of Uncertainty for Shipping Variation

S7.3 Uncertainty Analysis Results

Table S31: Uncertainty analysis Results - All Life Cycle Phases

Life Cycle Phase	Uncertainty Range (kg CO_2e/tonne)		
	Q1	This Study	Q3
**Diesel**	152.90	217.96	285.73
**Petrol**	2.62	3.74	4.87
**Aviation Fuel**	1.97	2.86	3.75
**Fertiliser**	5.23	5.48	13.66
**Pesticide**	17.10	26.92	27.23
**Electricity - Farm**	114.13	370.35	493.68
**Packaging**	63.43	64.05	67.93
**Transport by Road**	80.61	96.52	140.29
**Transport by Sea**	90.63	94.85	101.94
**Ripening Chamber**	10.70	21.71	46.18
**Carbon Sequestration**	-167.72	-521.88	-764.09
**Yield Variation**	-12.25	0.00	20.09
**Total**	359.36	382.58	441.27

S7.4 Example MATLAB Code to Determine Uncertainty with Monte Carlo Simulations

```
% Your provided data
data = [data point 1; data point 2; data point 3; etc];
% Fit a distribution to the data
pd = fitdist(data, 'Kernel');
% Generate random numbers from the fitted distribution
num_samples = 10000; % You can change this to your desired number of samples
samples = random(pd, num_samples, 1);
% Use Kernel Density Estimation to estimate the Probability Density Function (PDF)
[f,xi] = ksdensity(samples);
% Plot the estimated PDF
figure;
plot(xi,f);
hold on;
% Calculate and display measures of uncertainty
```

```
ci = prctile(samples, [2.5 97.5]); % 95% confidence interval
line1 = plot([ci(1), ci(1)], ylim, 'r--');
line2 = plot([ci(2), ci(2)], ylim, 'r--');
title('Estimated PDF from Monte Carlo Simulation');
xlabel(Insert Relevant X-Axis Label)');
ylabel('Probability Density');
legend([line1, line2], 'Lower bound of 95% CI', 'Upper bound of 95% CI');
% Create a new figure for the box plot
figure;
boxplot(samples);
title('Box Plot of Simulated Data');
xlabel(‘Insert Relevant Y-Axis Label’);
ylabel('Insert Relevant X-Axis Label');
% Calculate and print Q1 and Q3
q1 = prctile(samples, 25); % First quartile (Q1)
q3 = prctile(samples, 75); % Third quartile (Q3)
fprintf('First Quartile (Q1): %.2f\n', q1);
fprintf('Third Quartile (Q3): %.2f\n', q3);
```

S8. Projected Climate Change Impacts

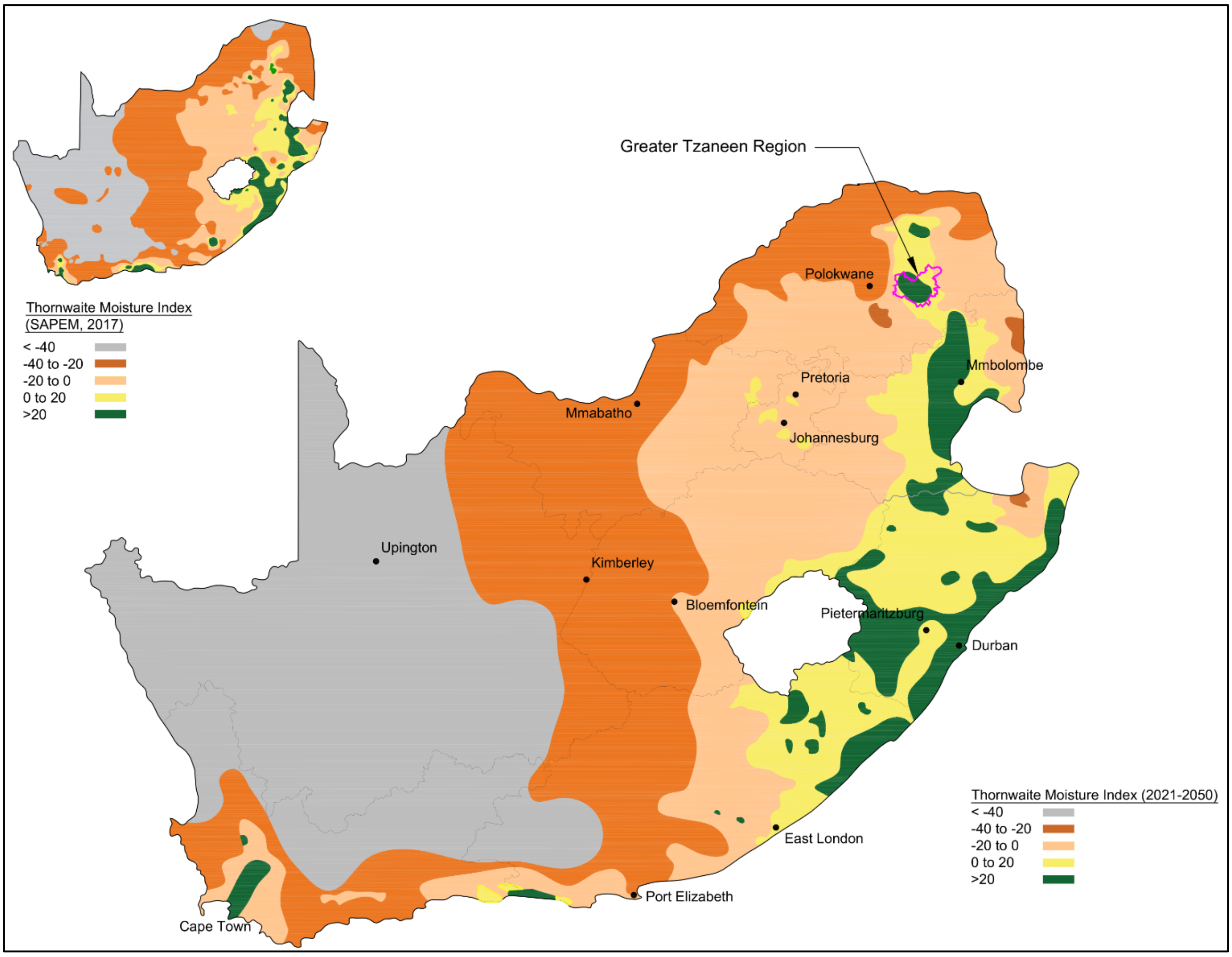

Figure S7: Thornthwaite Moisture Index changes for South Africa (2021–2050) (Blaauw et al., 2022)

S9. Comparison to Other Studies

Table S32: Comparison of avocado carbon footprint for different life cycle phases and on a per-calorie basis

**Life cycle phase**	**Farm**	**Processing and Packaging**	**Transport by Road**	**Transport by Ship**		**Ripening chamber**	**Carbon sequestration**	**Total (Excluding carbon sequestration)**	**Total (Including carbon sequestration)**	**On a per calorie basis**
**Unit**	kg CO_2e/tonne	kg CO_2e/tonne	g CO_2e/tonne-km	g CO_2e/TEU-km	g CO_2e/tonne-km	kg CO_2e/tonne	kg CO_2e/ha	kg CO_2e/tonne	kg CO_2e/tonne	g CO_2e/kcal
This study	***588.09***	***103.67***	***96.52***	***94.85***	***7.59***	***21.71***	***4 957.82***	***904.85***	***382.97***	***0.43***
**Du Plessis et al. (2022)***			86.08	190	18					
**Estee-Llorens et a., (2022)**								1090		
**Majumdar and McLaren (2023)**								430		
**NL – NIPHE (2021)**								1 315.00		
**Frankowska et al. (2019)**	1 056.00	192						2 400.00		
**Bell et al. (2018)**								450		
**Audsley et al. (2009)**								880		
**Stoessel et al. (2012)**								1247		
**Drewnowski et al. (2015)****										4.08
**Mærsk (2021)**					7.02					
**UK DoBEIS (2022a)**					13.08					
**Martin-Gorriz et al. (2020)*****							22 803.5		-600	

* Study relevant to the same geographical zone and supply chain

** Comparison against average of processed fruit

***Average for stone fruit

S10. Data Quality Matrix

Table S33: Data Quality Matrix (adapted from Blaauw et al., 2020)

Score	5 (Best)	4	3	2	1 (Worst)
**Reliability**	Verified data based on measurement	Verified data partly based on assumptions or non-verified data based on measurements	Non-verified data partly based on assumptions	Qualified estimate (e.g. by industry expert)	Non-qualified estimate
**Completeness**	Representative data from a sufficient sample of sites over an adequate period to even out normal fluctuations	Representative data from a smaller number of sites but for adequate periods	Representative data from an adequate number of sites but from shorter periods (i.e., less than 1 year)	Representative data but from a smaller number of sites and shorter periods or incomplete data from an adequate number of sites and periods	Representativeness unknown or incomplete data from a smaller number of sites and/or from shorter periods
**Temporal correlation – Age of data**	<= 3 years	<= 5 years	<= 10 years	<= 15 years	>= 15 years
**Geographical compatibility**	Data from South Africa or relevant to South African supply chain (including Netherlands, France and England)	UK, Europe, North American, Australia, New Zealand or World Average	Data from area with similar production conditions falling within the developing world.	-	All other countries and regions, including unknown
**Technological correction**	Data from enterprises (i.e. ZZ2 or Halls) and processes and fruit (i.e., avocado specific) under study	Data from processes and fruit under study but from different enterprises	Data relevant to processes and fruit under study but from different technology	Data on related processes or fruit (i.e., mango/banana) but same technology	Data on related processes or fruit but different technology

Table S34: Data Quality Indicator scores for indicators used in this study

DQI Number	DQI Reference	Reliability	Completeness	Temporal correlation – Age of data	Geographical compatibility	Technological correction	DQI Score (%)
I	Eskom (2021)	5	5	5	5	5	100%
II	EEA (2019)	5	5	5	5	5	100%
III	UK DoBEIS (2022a)	5	5	5	4	4	92%
IV	UK DoBEIS (2022b)	5	5	5	4	4	92%
V	ZZ2 (2015)	5	5	3	5	5	92%
VI	ZZ2 (2022)	5	5	5	5	5	100%
VII	Halls (2021)	5	5	5	5	5	100%
VIII	Broekman et al. (2020)	5	5	5	5	5	100%
IX	Gaidajis and Kakanis (2021)	5	4	5	4	2	80%
X	Audsley et al. (2009)	4	3	2	4	4	68%
XI	Blaauw and Maina (2021)	5	4	5	5	3	88%

XII	Albrecht & Kandji (2003)	4	4	1	4	4	68%
XIII	PE-Americas and Five Winds International (2010)	5	5	2	4	4	80%
XIV	Fitzgerald et al. (2011)	5	3	2	4	4	72%
XV	Nottenboom and Carriou (2009)	4	3	1	4	4	64%
XVI	Pedreschi et al. (2016)	4	4	3	3	4	72%
XVII	Worrell et al. (2000)	4	3	1	4	4	64%
XVIII	Eurotunnel (2022)	3	4	5	4	3	76%
XIX	Luo et al. (2006)	5	5	2	4	4	80%
XX	Zhou et al. (2022)	5	5	5	5	4	96%
XXI	Vergara and Silver (2019)	5	4	5	4	4	88%
XXII	Reincke Logistiek (2022)	3	4	5	5	3	80%

References:

These references are additions to the references provided in the main body text.

Aires, L.M.I., Pio, C.A., Pereira, J.S., 2007. Carbon dioxide exchange above a Mediterranean C3/C4 grassland during two climatologically contrasting years. Global Change Biology, Volume 14, Issue 3, pp. 539-555. https://doi.org/10.1111/j.1365-2486.2007.01507.x

Albrecht, A., Kandji, S.T., 2003. Carbon sequestration in tropical agroforestry systems. Agriculture, Ecosystems and Environment, Volume 99, pp. 15-27. https://doi.org/10.1016/S0167-8809(03)00138-5

Blengini, G.A., 2008. Using LCA to evaluate impacts and resources conservation potential of composting: A case study of the Asti District in Italy. Resources, Conservation and Recycling, Volume 52, Issue 12, pp. 1373-1381. https://doi.org/10.1016/j.resconrec.2008.08.002

Boerema, A., Van der Biest, K., Meire, P., 2016. Presentatio - Ecosystem services: Towards integrated marine infrastructure project assessment. https://www.researchgate.net/publication/310465854_Ecosystem_services_towards_integrated_marine_infrastructure_project_assessment

Boldrin, Al., Andersen, J.K., Møller, J., Christensen, T.H., Faviono, E., 2009. Composting and compost utilization: accounting of greenhouse gases and global warming contributions. Waste Management and Research: The Journal for a Sustainable Circular Economy, Volume 27, Issue 8. https://doi.org/10.1177/0734242X09345275

Brown, S., Kruger, C., Subler, S., 2008. Greenhouse gas balance for composting operations. Journal of Environmental Quality, Volume 37, Issue 4. https://doi.org/10.2134/jeq2007.0453

Buratti, C., Barbanera, M., Testarmata, F., Fantozzi, F., 2015. Life cycle assessment of organic waste management strategies: an Italian case study. Journal of Cleaner Production, Volume 89, pp. 125-136. http://dx.doi.org/10.1016/j.jclepro.2014.11.012

Castellani, F., Esposito, A., Geldermann, J., Altieri, R., 2019. Life cycle assessment of passively aerated composting in gas-permeable bags of olive mill waste. Life Cycle Assessment of Waste Management Systems, Volume 24, pp. 281-296. https://doi.org/10.1007/s11367-018-1514-0

Colón, J., Cadena, E., Pognani, M., Barrena, R., Sánchez, A., Font, X., Artola, A., 2012. Determination of the energy and environmental burdens associated with the biological treatment of source-separated Municipal Solid Waste. Energy & Environmental Science, Volume 5. http://dx.doi.org/10.1039/C2EE01085B

DeLonge, M.S., Ryals, R., Silver, W.L., 2013. A lifecycle model to evaluate carbon sequestration potential and greenhouse gas dynamics of managed grassland. Ecosystems, Volume 16, pp. 962-979. http://dx.doi.org/10.1007/s10021-013-9660-5

Drewnowski, A., Rehm, C.D., Martin, A., Verger, E.O., Voinnesson, M., Imbert, P., 2015. Energy and nutrient density of foods in relation to their carbon footprint. American Journal of Clinical Nutrition, Volume 101, Issue 1, pp. 184-191. https://doi.org/10.3945/ajcn.114.092486

Eurotunnel, 2022. Carbon-counter. https://www.eurotunnelfreight.com/uk/about/carbon-counter/

Halls, 2021. Maritime shipping data for the period 2020-2021. Personal communication.

IPCC, 2022. Intergovernmental Panel on Climate Change. Climate Change 2022: Mitigation of climate change. Working Group III Contribution to the Sixth Assessment Report of the IPCC. Geneva, Switzerland. Available online at https://report.ipcc.ch/ar6/wg3/IPCC_AR6_WGIII_Full_Report.pdf

Kato, T., Tang, Y., Gu, S., Hirota, M., Du, M., Li, Y., Zhao, X., 2006. Temperature and biomass influences on interannual changes in CO2 exchange in an alpine meadow on the Qinghai-Tibetan Plateau. Global Change Biology, Volume 12, Issue 7, pp. 1285-1298. https://doi.org/10.1111/j.1365-2486.2006.01153.x

Levis, J.W., Barlaz, M.A., 2011. What is the most environmentally beneficial way to treat commercial food waste? Environmental Science and Technology, Volume 45, Issue 17, pp. 7438-7444. https://doi.org/10.1021/es103556m

Liu, Z., Wang, X., Li, S., Bai, Z., Ma, L., 2022. Advanced composting technologies promotes environmental benefits and eco-efficiency: A life cycle assessment. Bioresource technology, Volume 346. https://doi.org/10.1016/j.biortech.2021.126576

López-Gálvez, F., Rasines, L., Conesa, E., Gómez, P.A., Artés-Hernández, F., Aguayo, E., 2021. Reusable Plastic Crates (RPCs) for Fresh Produce (Case Study on Cauliflowers): Sustainable Packaging but Potential Salmonella Survival and Risk of Cross-Contamination. Foods, Volume 10, Issue 6. https://doi.org/10.3390%2Ffoods10061254

Ma, S., Baldocchi, D.D., Xu, L., Hehn, T., 2007. Inter-annual variability in carbon dioxide exchange of an oak/grass savanna and open grassland in California. Agricultural and Forest Meteorology, Volume 147, Issue 3-4, pp. 157-171. https://doi.org/10.1016/j.agrformet.2007.07.008

Martínez-Blanco, J., Colón, J., Gabarrell, X., Font, X., Sánchez, A., Artola, A., Rieradevall, J., 2010. The use of life cycle assessment for the comparison of biowaste composting at home and full scale. Waste Management, Voluem 30, pp. 983-994. http://dx.doi.org/10.1016/j.wasman.2010.02.023

"Natural England Research Report, 2012. Carbon storage by habitat: Review of the evidence of the impacts of management decisions and condition of carbon stores and sources. Available online at: http://publications.naturalengland.org.uk/publication/1412347

Nordahl, S.L., Devkota, J.P., Amirebrahimi, J., Smith, S.J., Breunig, H.M., Preble, C.V., Satchwell, A.J., Jin, L., Brown, N.J., Kirchstetter, T.W., Scown, C.D., 2020. Life-cycle greenhouse gas emissions and human health trade-offs or organic waste management strategies. Environmental Science and technology, Volume 54, pp. 9200-9209. https://pubs.acs.org/doi/epdf/10.1021/acs.est.0c00364

Padeyanda, Y., Jang, Y.C., Ko, Y., Yi, S., 2015. Evaluation of environmental impacts of food waste management by material flow analysis (MFA) and life cycle assessment (LCA). Journal of Material Cycles and Waste Management, Volume 18, pp. 493-508. https://doi-org.uplib.idm.oclc.org/10.1007/s10163-016-0510-3

PE-Americas and Five Winds International, 2010. Corrugated packaging life-cycle assessment: Summary report. Corrugated Packaging Alliance. Illinois, USA. Available online at https://www.eco-conception.fr/data/sources/users/306/docs/corrugated-packaging-life-cycle-assessment.pdf

Pedreschi, R., Hollak, S., Harkema, H., Otma, E., Robledo, P., Westra, E., Somhorst, D., Ferreyra, R., Defilippi, B.G., 2016. Impact of postharvest ripening strategies on “Hass” avocado fatty acid profiles. South African Journal of Botany. Volume 103, pp. 32–35. https://doi.org/10.1016/j.sajb.2015.09.012

Pérez, T., Vergara, S.E., Silver, W.L., 2023. Assessing the climate change mitigation potential from food waste composting. Scientific Reports, Volume 13. https://www.nature.com/articles/s41598-023-34174-z.pdf

Pérgola, M., Persiani, A., Pastore, V., Palese, A.M., D'Adamo, C., De Falco, E., Celano, G., 2020. Sustainability assessment of the green compost production chain from agricultural waste: A case study in Southern Italy. Agronomy https://doi.org/10.3390/agronomy10020230

Phel, M., Arvesen, A., Humpenöder, Popp, A., Hertwich, E.G., Luderer, G., 2017. Understanding future emissions from low-carbon power systems by integration of life-cycle assessment and integrated energy modelling. Nature Energy, Volume 2, pp. 939-945. https://doi.org/10.1038/s41560-017-0032-9

Rana, R., Ganguly, R., Gupta, A.K., 2019. Life-cycle assessment of municipal solid-waste management strategies in Tricity region of India. Journal of Material Cycles and Waste Management, Volume 21, pp. 606-623. https://doi.org/10.1007/s10163-018-00822-0

Scott, R.L., Huxman, T.E., Williams, D.G., Goodrich, D.C., 2006. Ecohydrological impacts of woody-plant encroachment: seasonal patterns of water and carbon dioxide exchange within a semiarid riparian environment. Global Change Biology, Volume 12, Issue 2, pp. 311-324. https://doi.org/10.1111/j.1365-2486.2005.01093.x

Tessema, B., Sommer, R., Piikki, K., Söderström, M., Namirembe, S., Notenbaert, A., Tamene, L., Nyawira, S., Paul, B., 2020. Potential for soil organic carbon sequestration in grasslands in East African countries: A review. Grassland Science, Volume 66, Issue 3, pp. 135-144. https://doi.org/10.1111/grs.12267

UK DoBEIS, 2022a. United Kingdom Department of Business, Energy and Industrial Strategy. Greenhouse gas reporting: conversion factors 2022. UK DoBEIS. London, UK. Available

online at https://assets.publishing.service.gov.uk/government/uploads/system/uploads/attachment_data/file/1083854/ghg-conversion-factors-2022-condensed-set.xls

US EPA, 2016. United Stated Environmental Protection Agency. Documentation for Greenhouse Gas Emissions and Energy Factors Used in the Waste Reduction Model (WARM). Organic Materials Chapters. Office of Resource Conservation and Recovery. Washington D.C., USA. https://www.epa.gov/sites/default/files/2016-03/documents/warm_v14_organic_materials.pdf

Vågen, T.G., Lal, R., Singh, B.R., 2005. Soil carbon sequestration in sub-Saharan Africa: a review. Land Degradation and Development, Volume 16, Issue 1, pp. 53-71. https://doi.org/10.1002/ldr.644

Weligama Thuppahige, R.T., Gheewala, S.H., Babel, S., 2022. Environmental impact of organic fraction of municipal solid waste treatment by composting in Sri Lanka. Journal of Material Cycles and Waste Management, Volume 24, pp. 189-199. https://doi.org/10.1007/s10163-021-01305-5

WWF, 2021. World Wildlife Fund – UK. Driven to waste: The global impact of food loss and waste on farms. Available online at https://wwfeu.awsassets.panda.org/downloads/driven_to_waste___the_global_impact_of_food_loss_and_waste_on_farms.pdf

Yadav, P., Samadder, S.R., 2018. Environmental impact assessment of municipal solid waste management options using life cycle assessment: a case study. Environmental Science and Pollution Research, Volume 25, pp. 838-854. https://doi.org/10.1007/s11356-017-0439-7

Yay, A.S.E., 2015. Application of life cycle assessment (LCA) for municipal solid waste management: a case study of Sakarya. Journal of Cleaner Production, Volume 94, pp. 284-293. http://dx.doi.org/10.1016/j.jclepro.2015.01.089

Zhoa, L., Li, Y., Xu, S., Zhou, H., Gu, S., Yu, G., Zhao, X., 2006. Diurnal, seasonal and annual variation in net ecosystem CO2 exchange of an alpine shrubland on Qinghai-Tibetan plateau. Global Change Biology, Volume 12, Issue 10, pp. 1940-1953. https://doi.org/10.1111/j.1365-2486.2006.01197.x

ZZ2, 2015. ZZ2 Limpopo Province: Carbon footprint calculation 2014-2015. Personal communication.

ZZ2, 2022. Carbon footprint data for the period 2020-2021. Personal communication.
